# Granzyme B PET Imaging of Immune Checkpoint Inhibitor Combinations in Colon Cancer Phenotypes

**DOI:** 10.1007/s11307-020-01519-3

**Published:** 2020-07-23

**Authors:** J. L. Goggi, Y. X. Tan, S. V. Hartimath, B. Jieu, Y. Y. Hwang, L. Jiang, R. Boominathan, P. Cheng, T. Y. Yuen, H. X. Chin, J. R. Tang, A. Larbi, A. M. Chacko, L. Renia, C. Johannes, Edward G. Robins

**Affiliations:** 1 Singapore Bioimaging Consortium, Agency for Science, Technology and Research (A* STAR), 11 Biopolis Way, #01-02, Helios, 138667 Singapore; 2 Institute of Chemical and Engineering Sciences (ICES), A*STAR, 8 Biomedical Grove, #07, Neuros, 138665 Singapore; 3 Singapore Immunology Network, A*STAR, 8A Biomedical Grove, Immunos, 138648 Singapore; 4 Laboratory for Translational and Molecular Imaging (LTMI), Cancer and Stem Cell Biology Programme, Duke-NUS Medical School, 8 College Road, Singapore, 169857 Singapore; 5 p53 Laboratory, A*STAR, 8A Biomedical Grove, #06-04/05, Neuros/Immunos, 138665 Singapore; 6 Clinical Imaging Research Centre (CIRC), Yong Loo Lin School of Medicine, National University of Singapore, Singapore, 117599 Singapore

**Keywords:** Immune checkpoint inhibitor (ICI), [^18^F]AlF-*m*NOTA-GZP, CT26, MC38

## Abstract

**Purpose:**

Immune checkpoint inhibitor (ICI) monotherapy and combination regimens are being actively pursued as strategies to improve durable response rates in cancer patients. However, the biology surrounding combination therapies is not well understood and may increase the likelihood of immune-mediated adverse events. Accurate stratification of ICI response by non-invasive PET imaging may help ensure safe therapy management across a wide number of cancer phenotypes.

**Procedures:**

We have assessed the ability of a fluorine-labelled peptide, [^18^F]AlF-mNOTA-GZP, targeting granzyme B, to stratify ICI response in two syngeneic models of colon cancer, CT26 and MC38. *In vivo* tumour uptake of [^18^F]AlF-mNOTA-GZP following ICI monotherapy, or in combination with PD-1 was characterised and correlated with changes in tumour-associated immune cell populations.

**Results:**

[^18^F]AlF-*m*NOTA-GZP showed good predictive ability and correlated well with changes in tumour-associated T cells, especially CD8+ T cells; however, overall uptake and response to monotherapy or combination therapies was very different in the CT26 and MC38 tumours, likely due to the immunostimulatory environment imbued by the MSI-high phenotype in MC38 tumours.

**Conclusions:**

[^18^F]AlF-*m*NOTA-GZP uptake correlates well with changes in CD8+ T cell populations and is able to stratify tumour response to a range of ICIs administered as monotherapies or in combination. However, tracer uptake can be significantly affected by preexisting phenotypic abnormalities potentially confusing data interpretation.

**Electronic supplementary material:**

The online version of this article (10.1007/s11307-020-01519-3) contains supplementary material, which is available to authorized users.

## Introduction

Immune checkpoint receptors are crucial molecules that regulate the immune system, dampening T cell activation. Tumours exploit these checkpoints to evade the immune system. Immune checkpoint inhibitors (ICIs) have changed the landscape of cancer treatment; however, responses vary depending on tumour type and only a minority of patients show a durable response [1–4]. Combination ICI therapy is being actively pursued as a strategy to improve the rate of response. PD-1 and CTLA-4, the two most commonly studied checkpoints, are not functionally redundant and while they both act to modulate distinct populations of tumour-infiltrating lymphocytes (TILs), including CD4+, CD8+, regulatory T (T_reg_) cells, they do so with different mechanistic profiles. Thus, combination therapies may act in a complementary or synergistic fashion depending on the tumour microenvironment [5]. However, the biology surrounding combination therapies is not well understood and combined use may also increase the incidence of potentially severe side effects. Recent studies using a combined PD-1/CTLA-4 strategy have shown increased immune-related side effects and unexpected off target effects including cardiac toxicity [6]. It is hoped that newer ICIs may improve efficacy when combined with established inhibitors such as PD-1, while reducing unwanted side effects. The negative immune checkpoints TIM-3 (T cell immunoglobulin and mucin domain-3) and LAG-3 (lymphocyte-associated gene 3) are co-expressed with PD-1 on T cells and are being actively assessed in the clinic [7–9]. TIM-3 is expressed on numerous immune cell types including T cells, dendritic cells, and macrophages and is thought to be involved in immune tolerance by mediating T cell exhaustion [8]. LAG-3 is also a checkpoint modulator, is also thought to be involved in immune tolerance by mediating T cell exhaustion [7] and has been shown to be expressed on expressed on natural killer (NK), activated T cells and T_reg_ TILs [5, 10]. Alternative strategies include targeting OX40, a T cell co-stimulator expressed on CD4 and CD8 T cells during antigen specific priming which leads to T cell expansion and differentiation. OX40 antibody agonists have shown significant therapeutic efficacy both as monotherapies and in combination with PD-1 blockade leading to significant tumour regression [11]. Certain phenotypes, such as microsatellite instability (MSI), mutational burden, and neoantigenic load have been demonstrated to enrich response to PD-1 monotherapy [2, 12]. Currently, there is little evidence to determine whether these will also enrich response to the newer immune therapies and importantly no way to accurately predict individual patient response. Accurate stratification of treatment response to monotherapies and especially combination therapies, across all tumour phenotypes, is paramount to ensure safe and effective patient management.

Currently, there is a dearth of biomarkers capable of accurately stratifying ICI treatment response. Non-invasive imaging of the tumour microenvironment is being intensively researched to accurately assess immune therapies. Several studies have shown that TILs are necessary for checkpoint inhibitor efficacy; thus, radiopharmaceuticals have been developed to target T lymphocyte populations including CD3 and CD8. The presence of TILs alone, however, may not be enough to accurately stratify response due to immune cell regulation or tolerance [13–15]. Granzyme B is released upon activation of cytotoxic CD8+ T cells, providing information about T cell location and tumouricidal activity. A ^68^Ga-labelled peptide that targets GZB has been shown to reliably stratify response to ICIs in preclinical tumour models [16–18]. In the current study, we have developed and assessed a new fluorine-labelled derivative, [^18^F]AlF-mNOTA-GZP, to improve PET imaging sensitivity and spatial resolution [19]. The ability of [^18^F]AlF-mNOTA-GZP to stratify immunotherapy response was assessed in response to ICI monotherapy and combined ICI therapies in CT26 and MC38 syngeneic colon cancer models, frequently associated with MSI-low phenotype, and MSI-high phenotype [20], respectively. PET imaging results were then correlated to differences in TIL subpopulations by flow cytometry.

## Materials and Methods

### General

H-Asp(OtBu)-H NovaSyn TG resin (0.21 mmol/g) was obtained from Merck. Fmoc-amino acids, HATU and HOAt were obtained from Advanced Chemtech. Fmoc-glutamic acid was t-butyl protected. (p-SCN-Bn)-NOTA was purchased from Boc Sciences and Macrocyclics. Glacial acetic acid was purchased from JT Baker. Sep-Pak® light (46 mg) Accell™ plus QMA carbonate cartridges and Sep-Pak® C18 light cartridges were purchased from Waters Corporation. Saline solution (0.9 % *w*/*v*) was purchased from Braun Medical Industries. All other chemicals and reagents were purchased from Sigma-Aldrich, Fisher Scientific, and Tokyo Chemical Industry. No-carrier-added aqueous [^18^F]fluoride ion was produced *via* the [^18^O(p,n)^18^F] nuclear reaction (GE PETtrace 860 cyclotron). Quality control analytical radio-HPLC was performed on a UFLC Shimazdu HPLC system equipped with dual wavelength UV detector and a NaI/PMT-radiodetector (Flow-Ram, LabLogic). Radioactivity measurements were made with a CRC-55tPET dose calibrator (Capintec, USA).

### [^18^F]AlF-mNOTA-GZP Radiochemistry

NOTA–β-Ala–Gly–Gly–Ile–Glu–Phe–Asp–CHO (mNOTA-GZP) was synthesized using standard Fmoc chemistry and characterized by HPLC and mass spectroscopy as previously described (see Supplemental Materials, Table S1) [18]. Aqueous [^18^F]fluoride (typically 10 GBq in 2.3 mL) was trapped on a Sep-Pak® light (46 mg) Accell™ plus QMA carbonate cartridge (pre-conditioned with 10 mL water) and washed with a further 5 mL of water. The trapped [^18^F]fluoride anion was eluted with 0.9 % *w*/*v* saline (0.2 mL) and adjusted to pH 4 with glacial acetic acid (1 μL). To the pH-adjusted [^18^F]fluoride solution was added 2 mM AlCl_3_ in 0.1 M pH 4 NaOAc buffer (24 μL) and ethanol (0.2 mL). This solution was then added into a reaction vial containing mNOTA-GZP (0.1 mg). The reaction vial was sealed and heated at 100 °C for 15 min without stirring. After cooling to room temperature, the crude reaction mixture was diluted with water (40 mL) and loaded on a Sep-Pak® C18 light cartridge (pre-conditioned with 5 mL ethanol and 10 mL water). The cartridge was washed with a further 5 mL of water. [^18^F]AlF-mNOTA-GZP was eluted with 70 % ethanol in water (0.4 mL), and diluted with 0.9 % *w*/*v* saline to a final concentration of 10 % ethanol in saline. The radiochemical purity of [^18^F]AlF-mNOTA-GZP was assessed by analytical radio-HPLC (Luna Phenomenex C18(2), 5 μm, 100 Å, 250 mm × 4.6, 0.05 M NH_4_OAc pH 4.5 (solvent A) and acetonitrile (solvent B), gradient elution 0–6 min 10 % to 95 % B then 6–11 min 95 % B, flow rate 1 mL/min, column temperature 30 °C, λ = 254 nm). The retention time of mNOTA-GZP and [^18^F]AlF-mNOTA-GZP was between 7.1 to 7.2 min. [^18^F]AlF-mNOTA-GZP was obtained as a colourless solution in 10 % ethanol in saline (pH = 7.4) with a non-decay corrected radiochemical yield of 17–25 % (total reaction and purification time 50 min), with a radiochemical purity of 98–99 % and molar activity 45–90 GBq/μmol (*n* = 6).

### Animal Procedures

All animal procedures were carried out in accordance with the Institutional Animal Care and Use Committee Singapore (IACUC No. 181399) and conformed to the US National Institutes of Health (NIH) guidelines and public law. Balb/c and C57BL/6 mice aged 6–8 weeks were purchased from In Vivos Singapore, were kept at room temperature with a 12-h light-dark cycle and had free access to food and water. The murine colon tumour cell lines, CT26 and MC38, were acquired from ATCC and cultured in RPMI/ DMEM supplemented with 10 % foetal bovine serum, 100 U/mL penicillin and 100 μg/mL streptomycin, at 37 °C in a humidified atmosphere at 5 % CO_2_. CT26 or MC38 cells (1 × 10^6^) were prepared in a 1:1 (*v*/*v*) ratio in Matrigel (Sigma) and injected subcutaneously into the right shoulder of Balb/c mice or C57BL/6 mice, respectively. Rat IgG2a anti-mouse PD-1 (⍺PD1 mAb RMP1–14), mouse IgG2b anti-mouse CTLA-4 (⍺CTLA4 mAb 9D9), rat IgG1 anti-mouse OX40 (⍺OX40 mAb OX-86), rat IgG2a anti-mouse TIM-3 (⍺TIM3 mAb RMT3–23), rat IgG1 anti-mouse LAG-3 (⍺LAG3 mAb C9B7W), and rat IgG2a isotype control (⍺-trinitrophenol mAb), were purchased from Bio-X Cell. All mice were treated by intraperitoneal (i.p.) injection of either control IgG (5 mg/kg) or monotherapy (⍺PD1 (10 mg/kg), ⍺CTLA4 (5 mg/kg), ⍺OX40 (5 mg/kg), ⍺TIM3 (5 mg/kg) or ⍺LAG3 (10 mg/kg)) or combination therapy (⍺CTLA4 (5 mg/kg), ⍺OX40 (5 mg/kg), ⍺TIM3 (5 mg/kg) or ⍺LAG3 (10 mg/kg) in combination with ⍺PD1 (10 mg/kg)) on days 6, 9 and 12 following tumour inoculation (in order to maintain treatment efficacy ⍺OX40 injected 24 h before ⍺PD1 when used in combination). *In vivo* subcutaneous tumour lengths were measured by callipers on days 5, 8, 12, and 14 after tumour inoculation. Tumour volume was then calculated using the modified ellipsoid formula 1/2(length × width^2^) [21]. Tumour growth inhibition (%TGI) was determined using the formula TGI(%) = (Vc-Vt)/(Vc-Vo) × 100, where Vc and Vt are the mean of control and treated groups at the end of the study and Vo at the start. Successful tumour response was defined as greater than 50 % tumour growth inhibition, allowing differentiation between treatment responders (TR, tumours with greater than 50 % tumour growth inhibition) and treated non-responders (TNR, an aggregate of tumours with less than 50 % tumour growth inhibition across all treatment arms).

### PET-CT/MR Imaging

Animals were imaged 14 days after treatment initiation using a Siemens Inveon PET-CT and a Mediso nanoscan 3 T MR-PET. Briefly, animals were anaesthetised using inhalational isoflurane (maintained at 1.5 % alveolar concentration) and injected with [^18^F]AlF-mNOTA-GZP (~ 10 MBq) *via* the lateral tail vein. Static PET acquisitions were performed at 60–80 min post-injection (p.i.) and CT and MRI scans were used for co-registration. Animals were monitored for maintenance of body temperature and respiration rate during imaging studies using the Biovet physiological monitoring system. Post-analysis of reconstructed calibrated images were performed with FIJI and Amide software (version 10.3 Sourceforge). Uptake of radioactivity in the tumour was determined by the placement of a volume of interest (VOI) delineated by CT and MR imaging. A VOI was also placed in the quadriceps muscle to provide reference tissue values. Data are expressed as % of the injected dose per gram (%ID/g) of tumour tissue in the VOI.

### Fluorescence-Assisted Cell Sorting (FACS) and Analysis

Tumours were excised after *in vivo* PET imaging on day 14 and immediately processed for flow cytometry. A single-cell suspension was generated by incubating in modified RPMI (Gibco) supplemented with 10 % heat-inactivated foetal bovine serum (Gibco, Life Technologies), 20 μg/ml DNAse1 (Sigma-Aldrich) and 200 μg/ml Collagenase (Sigma-Aldrich). The samples were mechanically diced and incubated for 1 h at 37 °C and dissociated into single cells by passing through a 100 μm cell strainer. The samples were then counted and assessed for viability with Trypan Blue (Sigma-Aldrich). Cells were stained with antibodies against CD45 (clone 30-F11 BV570; Biolegend), CD3 (clone 500A2 BUV563; BD Biosciences), CD4 (clone RM4-5 BV650; BD Biosciences), CD8 (clone 53-6.7 BV510; BD Biosciences), CD25 (clone PC61 BUV395; BD Biosciences), F4/80 (clone BM8 biotin; Biolegend), CD206 (clone C068C2 PE-Cy7; Biolegend), CD68 (clone FA-11 PE-Cy7; Biolegend), Ly6C (clone HK1.4 BV605; Biolegend), NKp46 (clone 29A1.4 BUV737; BD Biosciences), CD11b (clone M1/70 APC-Cy7; Biolegend), I-A/I-E (clone M5/114.15.2 BV785; Biolegend), Ly6G (clone 1A8 BV480; BD Biosciences), FoxP3 (clone 150D AlexaFluor647; Biolegend), Fixable Live/Dead Blue (Invitrogen), Streptavidin BUV805 (BD Biosciences), PD-L1 (clone MIH5 BV421; BD Biosciences), CD107a (clone 1D4B FITC; Biolegend), CD170 (clone E50-2440 PECF594; BD Biosciences), Perforin (clone S16009A PE; Biolegend), CD11c (clone N418 BV711, Biolegend), and Granzyme B (GZB clone QA16A02 AlexaFluor700; Biolegend). Flow cytometry was performed on a BD FACSymphony. Fluorophore compensations and detector voltage were set up using single stains on murine spleen cells. Data was recompensated and analysed using FlowJo V10.5 software (FlowJo LLC).

### Statistical Analysis

Data were analysed using a Kruskal-Wallis 1-way ANOVA with a Dunn’s post-test using GraphPad Prism version 8.0.0 for Windows, GraphPad Software, San Diego, CA, USA, www.graphpad.com; *P* < 0.05 was considered statistically significant. Data are expressed as mean ± SD unless otherwise indicated.

## Results

### Radiochemistry Synthesis and *In Vitro* Characterisation of [^18^F]AlF-mNOTA-GZP

[^18^F]AlF-mNOTA-GZP was efficiently prepared by adaptation of the peptide labelling method described by McBride et al. in 2009 [22]. Starting from aqueous [^18^F]fluoride, [^18^F]AlF-mNOTA-GZP was obtained in a non-decay-corrected radiochemical yield of 17–25 % as a formulated product suitable for injection (10 % ethanol in saline, pH = 7.4) in a total reaction time of 50 min with a radiochemical purity of 98–99 % and molar activity 45–90 GBq/μmol. Similarly, [^19^F]AlF-mNOTA-GZP reference material was prepared using a similar synthetic procedure (see supplementary materials) and isolated as a 0.25 mg/mL solution of [^19^F]AlF-mNOTA-GZP in 10 % ethanol in saline. *In vitro*, [^19^F[AlF-mNOTA-GZP retained the expected enzyme inhibition efficiency of its’ [^68^Ga]gallium-labelled equivalent [17], with an inhibition potency of K_i_ ~ 78 ± 36 nM (Supplementary Materials, Fig. S1).

### Assessment of Treatment Efficacy Using Tumour Growth Inhibition

**Fig. 1:**
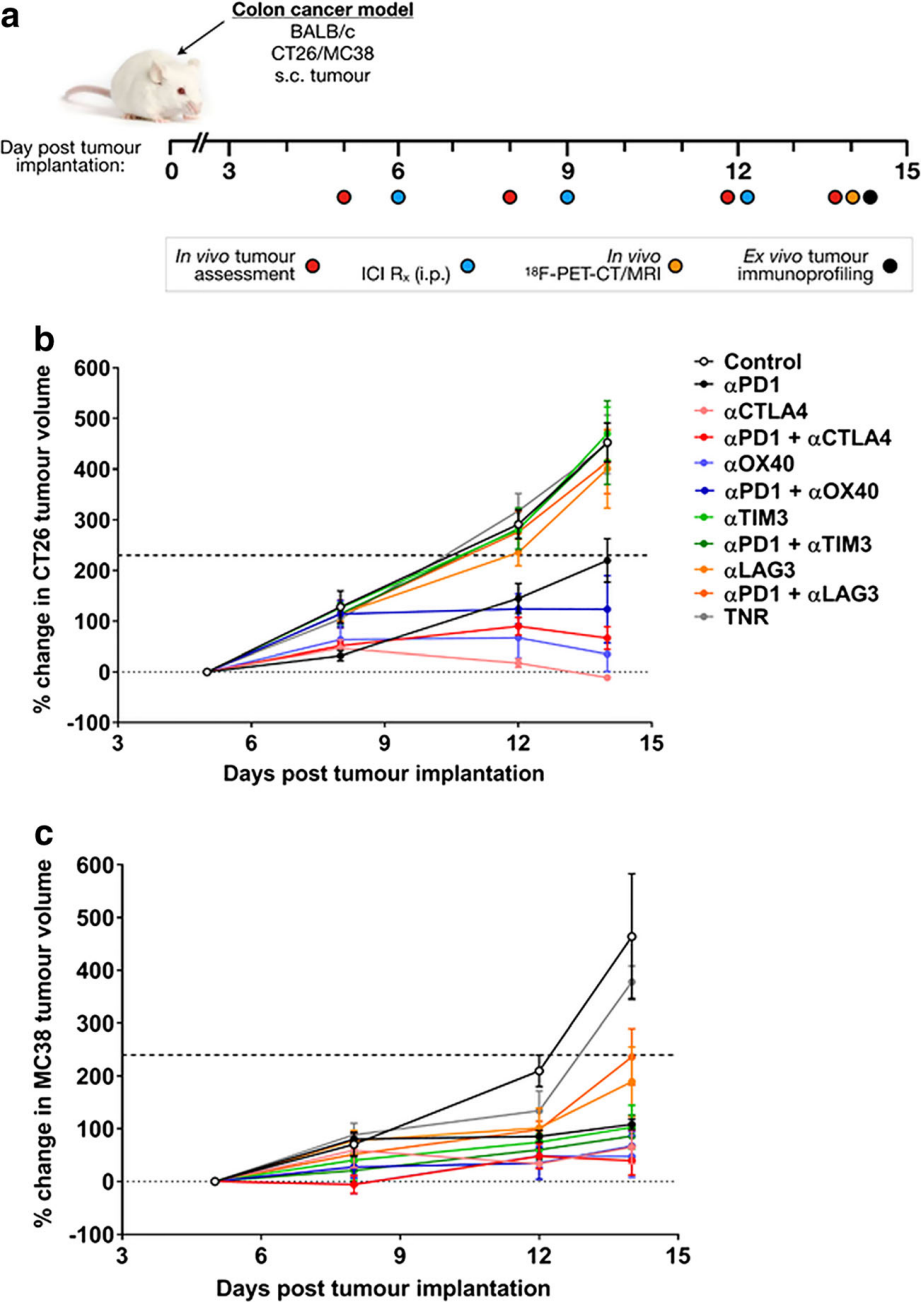
Comparison of immunotherapeutic effect of ICIs on change in tumour volume. **a** Schematic representation of timeline shows dosing regimen. Mice (*n* = 10) were i.p. treated with control IgG, monotherapies or combinations with αPD1 on days 6, 9 and 12 post-tumour implantation. Tumour size was measured on days 5, 8, 12 and 14 post-tumour implantation. **b** CT26 tumour-bearing mice and **c** MC38 tumour-bearing mice. Data are represented as % change in tumour volume from first day of assessment and are indicated as mean ± SD (*n* = 7–10). TNR, treated non-responder, 50 % tumour growth inhibition shown as a dotted line.

Balb/c mice bearing CT26 and C57BL/6 mice bearing MC38 colon tumours were treated with either control IgG, an ICI monotherapy regime (either ⍺PD1, ⍺CTLA4, ⍺OX40, ⍺TIM3 or ⍺LAG3 alone) or an ICI combination regime (⍺CTLA4, ⍺OX40, ⍺TIM3 or ⍺LAG3 combined with ⍺PD1) as shown in Fig. 1a. Initial tumour volume assessment at day 5 after tumour cell inoculation showed similar tumour volumes ~ 100 mm^3^ for the two tumour types. However, the CT26 and MC38 colon tumours showed very different responses to ICI monotherapy or combined ICI therapies as determined by tumour growth inhibition, as shown in Figs. 1b and c and Supplemental Tables S2 and S3.

### Assessment of Treatment Efficacy Using [^18^F]AlF-mNOTA-GZP PET Imaging

**Fig. 2:**
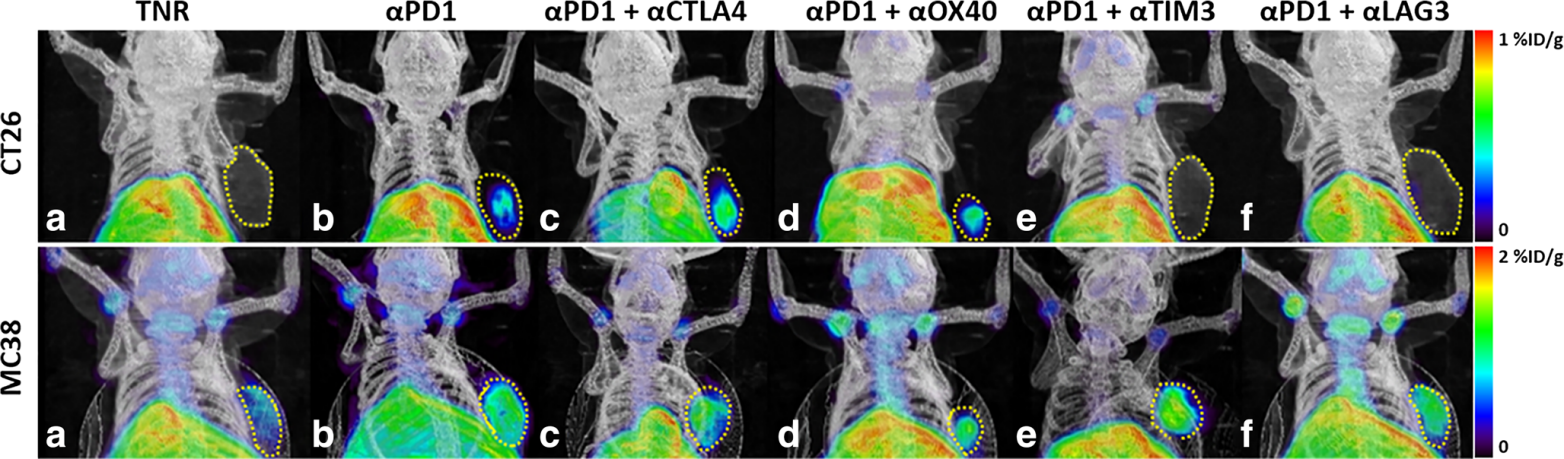
Representative maximum intensity projection PET/CT images of [^18^F]AlF-mNOTA-GZP tumour uptake in CT26 tumours (upper panel) and MC38 tumours (lower panel) following ICI monotherapy, and combination therapies for (a) treated non-responders (TNR), (b) ⍺PD1 monotherapy, (c) combination ⍺PD1 and ⍺CTLA4, (d) ⍺PD1 and ⍺OX40, (e) ⍺PD1 and ⍺TIM3 therapy and (f) ⍺PD1 and ⍺LAG3 therapy. Yellow dashed line indicates tumour boundary. Mice administered ~ 10 MBq [^18^F]AlF-mNOTA-GZP, and images acquired from 60 to 80 min post-tracer injection.

**Fig. 3:**
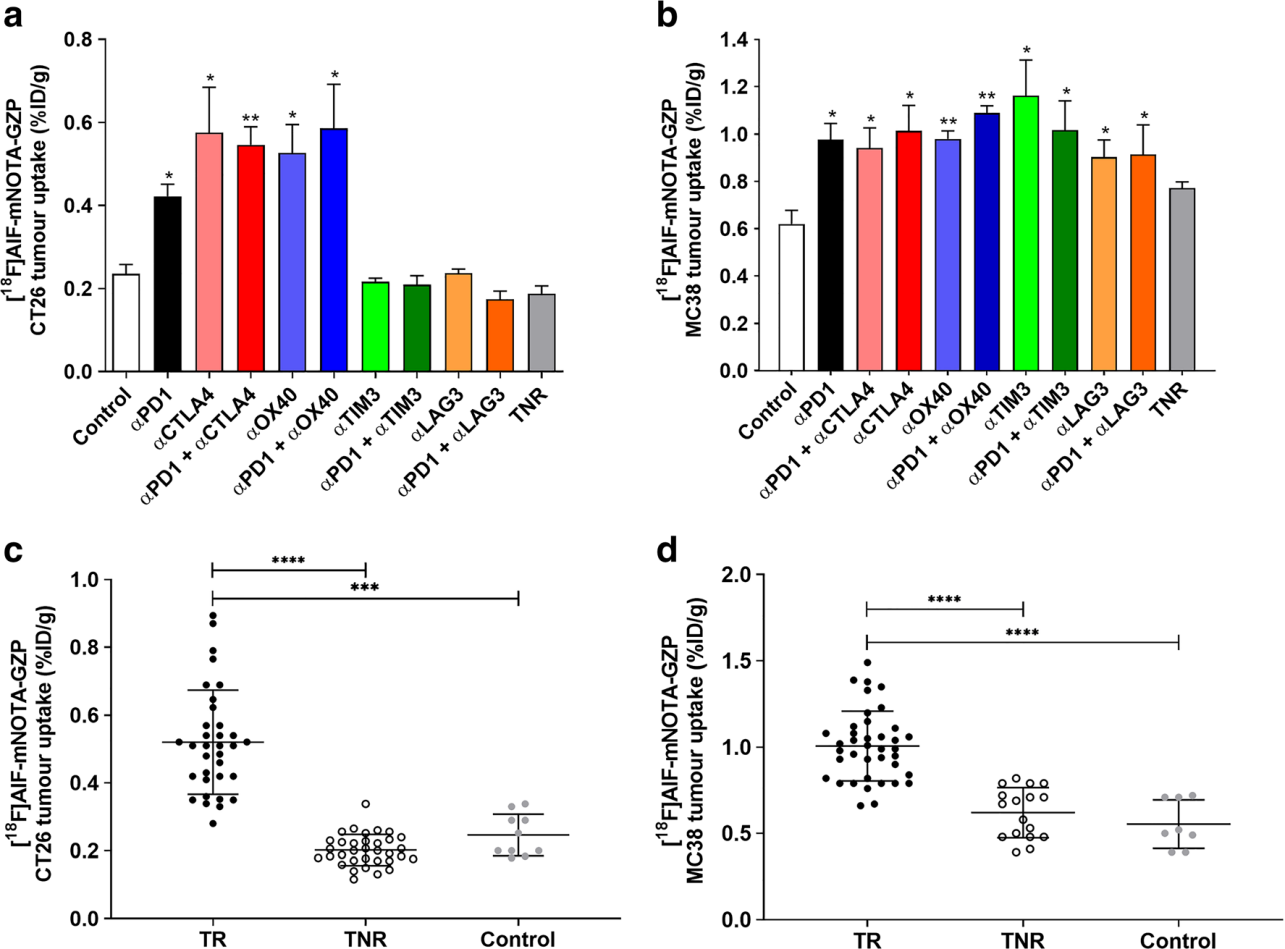
*In vivo* assessment of [^18^F]AlF-mNOTA-GZP tumour uptake from PET-CT/MRI defined volumes of interest (VOI) from individual mice subjected to ICI treatment. **a** For CT26 tumour-bearing animals, significant increases in [^18^F]AlF-mNOTA-GZP tumour uptake was observed between responders in treatment arms with ⍺PD-1, ⍺CTLA4, or ⍺OX40 monotherapy, and combined therapies of ⍺PD1 with ⍺CTLA4, and ⍺PD1 with ⍺OX40, compared to either control treated animals or treated non-responders (TNR) (*n* = 5–10; *P < 0.05; **P < 0.01, with data shown as mean %ID/g ± SEM). **b** In MC38 tumour-bearing mice, significant increases in [^18^F]AlF-mNOTA-GZP tumour uptake was observed between responders in all monotherapy and combination therapy arms compared to non-responders or control treated animals (*n* = 5–10; *P < 0.05; **P < 0.01, with data shown as mean %ID/g ± SEM). **c** [^18^F]AlF-mNOTA-GZP tumour uptake in CT26 TRs, TNRs and controls (***P < 0.001, ****P < 0.0001, data shown as individual %ID/g ± SD). **d** [^18^F]AlF-mNOTA-GZP tumour uptake in MC38 TRs, TNRs and controls (****P < 0.0001, data shown as individual %ID/g ± SD).

[^18^F]AlF-mNOTA-GZP PET imaging showed adequate tumour-to-background contrast for tumour visualisation in the two tumour models assessed. [^18^F]AlF-mNOTA-GZP showed heterogeneous *in vivo* tumour tracer uptake across the different treatment arms in each of the tumour models (Fig. 2). Quantitative tumour VOI analysis from PET-CT/MRI images at 60–80 min p.i. showed that [^18^F]AlF-mNOTA-GZP is able to detect significant differences in tracer uptake between the different treatment arms and was able to differentiate responders from treated non-responders (TNR, an aggregate of non-responders across all treatment arms) or control treatment in the CT26-tumour bearing mice (Table 1, Fig. 3a, c).

**Table 1:** [^18^F]AlF-mNOTA-GZP tumour uptake from PET-CT/MRI-defined volumes of interest (VOI) from individual mice bearing either CT26 or MC38 tumours subjected to ICI treatment. Data are shown as mean %ID/g ± S.D. of control groups, treatment responders (TR) across individual treatment arms, and all treatment non-responders (TNR) (*n* = 5–10 mice/group; *P < 0.05; **P < 0.01 comparing TR to TNR, ^$^*P* < 0.05; ^$$^*P* < 0.01 comparing TR to control)

	[^18^F]AlF-*m*NOTA-GZP tumour uptake	
	CT26	MC38
Control	0.23 ± 0.06	0.62 ± 0.14
Treatment responders (TR)		
αPD1	0.42 ± 0.09*^$^	0.98 ± 0.17*^$^
αCTLA4	0.58 ± 0.24*^$^	0.94 ± 0.21*^$^
αPD1 + αCTLA4	0.55 ± 0.10**^$$^	1.01 ± 0.24*^$$^
αOX40	0.53 ± 0.14*^$^	0.98 ± 0.07**^$$^
αPD1 + αOX40	0.59 ± 0.24*^$^	1.09 ± 0.06**^$$^
αTIM3	0.22 ± 0.02	1.16 ± 0.34*^$^
αPD1 + αTIM3	0.21 ± 0.05	1.02 ± 0.25*^$^
αLAG3	0.24 ± 0.02	0.90 ± 0.13*^$^
αPD1 + αLAG3	0.17 ± 0.04	0.92 ± 0.18*^$^
Treatment non-responders (TNR)	0.19 ± 0.06	0.77 ± 0.06

Low [^18^F]AlF-mNOTA-GZP uptake was observed in the control antibody treatment group (0.23 ± 0.06 %ID/g) and TNRs (0.19 ± 0.06 %ID/g) as well as in the treatment groups that did not inhibit tumour growth (⍺LAG3: 0.24 ± 0.02 %ID/g; ⍺PD1 + ⍺LAG3: 0.17 ± 0.04 %ID/g; ⍺TIM3: 0.22 ± 0.02 %ID/g; and ⍺PD1 + ⍺TIM3: 0.21 ± 0.05 %ID/g). However, significantly higher uptake was observed in treatment groups that successfully inhibited tumour growth including ⍺PD1 alone (0.42 ± 0.09%ID/g; *P < 0.05), ⍺CTLA4 alone (0.58 ± 0.24%ID/g; *P < 0.05), combined ⍺PD1 + ⍺CTLA4 (0.55 ± 0.10%ID/g; **P < 0.01), ⍺OX40 alone (0.53 ± 0.14%ID/g; *P < 0.05), and combined ⍺PD1 + ⍺OX40 (0.59 ± 0.24%ID/g; *P* < 0.05).

Likewise imaging with [^18^F]AlF-mNOTA-GZP reliably differentiated between responders and non-responders or control treatment in the MC38 tumour-bearing animals (Table 1, Fig. 3b).

Overall higher [^18^F]AlF-mNOTA-GZP uptake was observed in the MC38 tumours. Higher background uptake was observed in the control antibody treated group (0.62 ± 0.14%ID/g) and TNRs (0.77 ± 0.06%ID/g) compared to CT26 tumours. However, even greater uptake was observed in the MC38 tumours in treatment groups that responded to therapy including ⍺PD1 alone (0.98 ± 0.17%ID/g; *P < 0.05), ⍺CTLA4 alone (0.94 ± 0.21%ID/g; *P < 0.05), combined ⍺PD1 + ⍺CTLA4 therapy (1.01 ± 0.24%ID/g; *P < 0.05), ⍺OX40 alone (0.98 ± 0.07%ID/g; **P < 0.01), combined ⍺PD1 + ⍺OX40 therapy (1.09 ± 0.06%ID/g; **P < 0.01), ⍺TIM3 alone (1.16 ± 0.34%ID/g; *P < 0.05), combined ⍺PD1 + ⍺TIM3 therapy (1.02 ± 0.25%ID/g; *P < 0.05), ⍺LAG3 monotherapy (0.90 ± 0.13%ID/g; *P < 0.05) and combined ⍺PD1 + ⍺LAG3 therapy (0.92 ± 0.18%ID/g; *P < 0.05). Response evaluation across all treatment arms showed that [^18^F]AlF-mNOTA-GZP was significantly higher for responders than non-responders in CT26 tumours (median uptake 0.51 *vs* 0.21, *P* < 0.0001) (Fig. 3c) and MC38 tumours (median uptake 0.99 *vs* 0.67, *P* < 0.0001) (Fig. 3d).

### [^18^F]AlF-mNOTA-GZP Tumour Uptake Correlates with FACS-Defined Immune Profile

**Fig. 4:**
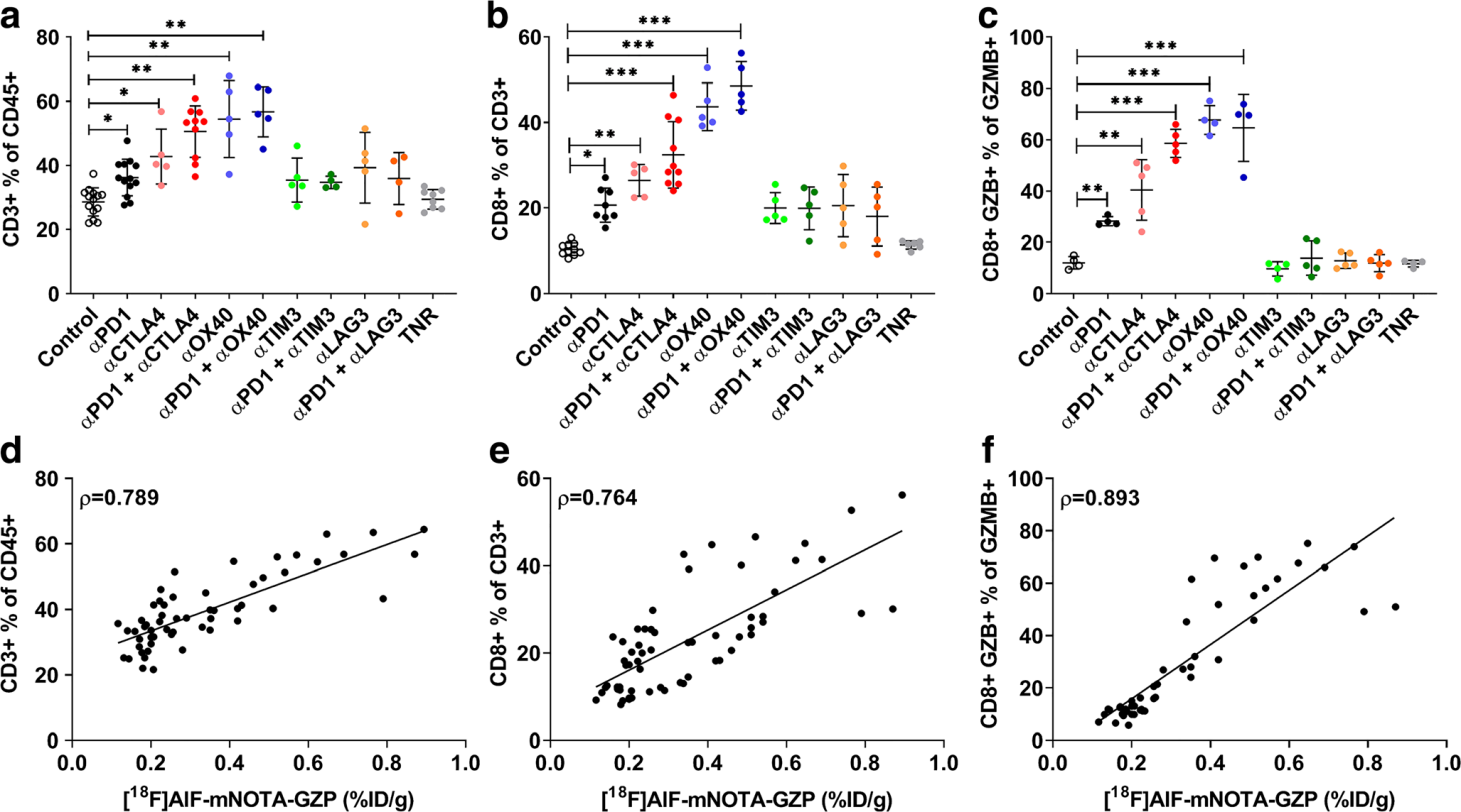
FACS analysis of tumour-infiltrating leukocyte (TIL) populations from CT26 tumour-bearing mice at day 14 post-induction of ICI monotherapy or combination therapies. Percentages of (a) CD3+ T cells relative to CD45+ T cells, (b) CD8+ TILS relative to total CD3+ TILS, and (c) CD8+ GZB+ TILS relative to total GZB+ cells across all treatment arms. Data are shown as individual values with mean ± S.D. and are representative of n = 5–10 mice/ group. * *P* < 0.05; ** *P* < 0.01; *** *P* < 0.001. (d-f) Tumour uptake of [^18^F]AlF-mNOTA-GZP (%ID/g) plotted against paired values for relative abundance of TILs gated for (d) CD3+, (e) CD8+, and (f) CD8+ GZB cells. A linear regression model of tracer uptake *versus* individual TILs is shown, along with Spearman’s correlation (*ρ*) and corresponding *P* values. Spearman’s *ρ* show strongest correlation between [^18^F]AlF-mNOTA-GZP tumour uptake and CD8+ GZB+ cells TILs.

To quantitate the changes in CT26 TILs in response to therapy, we analysed the distribution of different immune cell markers (Table 2, Fig. 4a–c). Significant differences were only observed when assessing T cell populations, including the total T cell population (marked by CD3+ relative to the CD45+ lymphocyte marker) and cell numbers associated with T cell effector function (namely with CD8+ T cells and GZB+ CD8+ T cells).

**Table 2:** FACS analysis of tumour-infiltrating leukocyte (TIL) populations from CT26 tumour-bearing mice at day 14 post-induction of ICI monotherapy or combination therapies. Percentages of T cell subpopulations across control groups, treatment responder (TR) arms, and all treatment non-responders (TNR) across all treatment arms. Data are shown as mean % of cells ± S.D. and are representative of *n* = 5–10 mice/ group, *P < 0.05; **P < 0.01, ***P < 0.001, comparing TR to TNR

	CT26		
	CD3+ % of CD45+	CD8+ % of CD3+	CD8+ GZB+ % of GZB+
Control	28.6 ± 4.4	10.4 ± 1.0	12.1 ± 2.4
TR			
αPD1	36.2 ± 5.7*	20.7 ± 4.0*	28.2 ± 1.8**
αCTLA4	42.8 ± 8.6*	26.5 ± 3.7**	40.4 ± 11.8**
αPD1 + αCTLA4	50.6 ± 8.0**	32.4 ± 7.8***	58.6 ± 5.5***
αOX40	54.5 ± 12.0**	43.7 ± 5.6***	67.8 ± 5.6***
αPD1 + αOX40	56.7 ± 7.8**	48.6 ± 5.7***	64.7 ± 13.1**
αTIM3	35.4 ± 6.9	19.0 ± 3.9	9.7 ± 2.8
αPD1 + αTIM3	34.7 ± 4.9	19.9 ± 5.0	13.9 ± 6.7
αLAG3	39.3 ± 11.0	20.6 ± 7.3	12.9 ± 3.0
αPD1 + αLAG3	35.9 ± 8.1	18.0 ± 6.9	12.0 ± 3.3
TNR	29.4 ± 3.1	11.4 ± 0.9	11.8 ± 1.3

Significant increases in CD3+ infiltration was observed in responders to ⍺PD1 monotherapy (36.2 ± 5.7; *P < 0.05), ⍺CTLA4 monotherapy (42.8 ± 8.6; *P < 0.05) and ⍺OX40 monotherapy (54.5 ± 12.0; **P < 0.01) as well as ⍺PD1 + ⍺CTLA4 combination therapy (50.6 ± 8.0; **P < 0.01) and ⍺PD1 + ⍺OX40 combination therapy (56.7 ± 7.8; *P < 0.01) compared to control treatment (28.6 ± 4.4) and TNRs (29.4 ± 3.1); however, no significant increases were observed in ⍺TIM3 monotherapy (35.4 ± 6.9), ⍺LAG3 monotherapy (39.3 ± 11.0), ⍺PD1 + ⍺TIM3 combination therapy (34.7 ± 1.9) or ⍺PD1 + ⍺LAG3 combination therapy (35.9 ± 8.1; as a % of CD45+ cells).

Significant increases in CD8+ infiltration were observed in responders to ⍺PD1 monotherapy (20.7 ± 4.0; *P < 0.05), ⍺CTLA4 monotherapy (26.5 ± 3.7; **P < 0.01) and ⍺OX40 monotherapy (43.7 ± 5.6; ***P < 0.001) as well as ⍺PD1 + ⍺CTLA4 combination therapy (32.4 ± 7.8; ***P < 0.001) and ⍺PD1 + ⍺OX40 combination therapy (48.6 ± 5.7; ***P < 0.001) compared to control treatment (10.4 ± 1.0) and TNRs (11.4 ± 0.9). Again, no significant increases were observed in ⍺TIM3 monotherapy (19.0 ± 3.6), ⍺LAG3 monotherapy (20.6 ± 7.3), ⍺PD1 + ⍺TIM3 combination therapy (19.9 ± 5.0) or ⍺PD1 + ⍺LAG3 combination therapy (18.0 ± 6.9; as a % of CD3+ cells).

Significant increases in CD8+ GZB+ cell infiltration were also observed in responders to ⍺PD1 monotherapy (28.2 ± 1.8; **P < 0.01), ⍺CTLA4 monotherapy (40.4 ± 11.8; **P < 0.01) and ⍺OX40 monotherapy (67.8 ± 5.6; ***P < 0.001) as well as ⍺PD1 + ⍺CTLA4 combination therapy (58.6 ± 5.5; ***P < 0.001) and ⍺PD1 + ⍺OX40 combination therapy (64.7 ± 13.1; ***P < 0.01) compared to control treatment (12.1 ± 2.4) or TNRs (11.8 ± 1.3). No significant differences were observed in ⍺TIM3 monotherapy (9.7 ± 2.8), ⍺LAG3 monotherapy (12.9 ± 3.0), ⍺PD1 + ⍺TIM3 combination therapy (13.9 ± 6.7) or ⍺PD1 + ⍺LAG3 combination therapy (12.0 ± 3.3; as a % of GZB+ cells).

Correlation analysis of [^18^F]AlF-mNOTA-GZP tumour uptake with TILs was performed in CT26 tumours. PET imaging showed a positive association with CD3+ T cells (Fig. 4d, *ρ* = 0.789; *P* < 0.0001), and CD8+ T cells (Fig. 4e, *ρ* = 0.764; *P* < 0.0001). The strongest correlation was observed with GZB+ CD8+ T cells (Fig. 4f, *ρ* = 0.893; *P* < 0.0001).

**Fig. 5:**
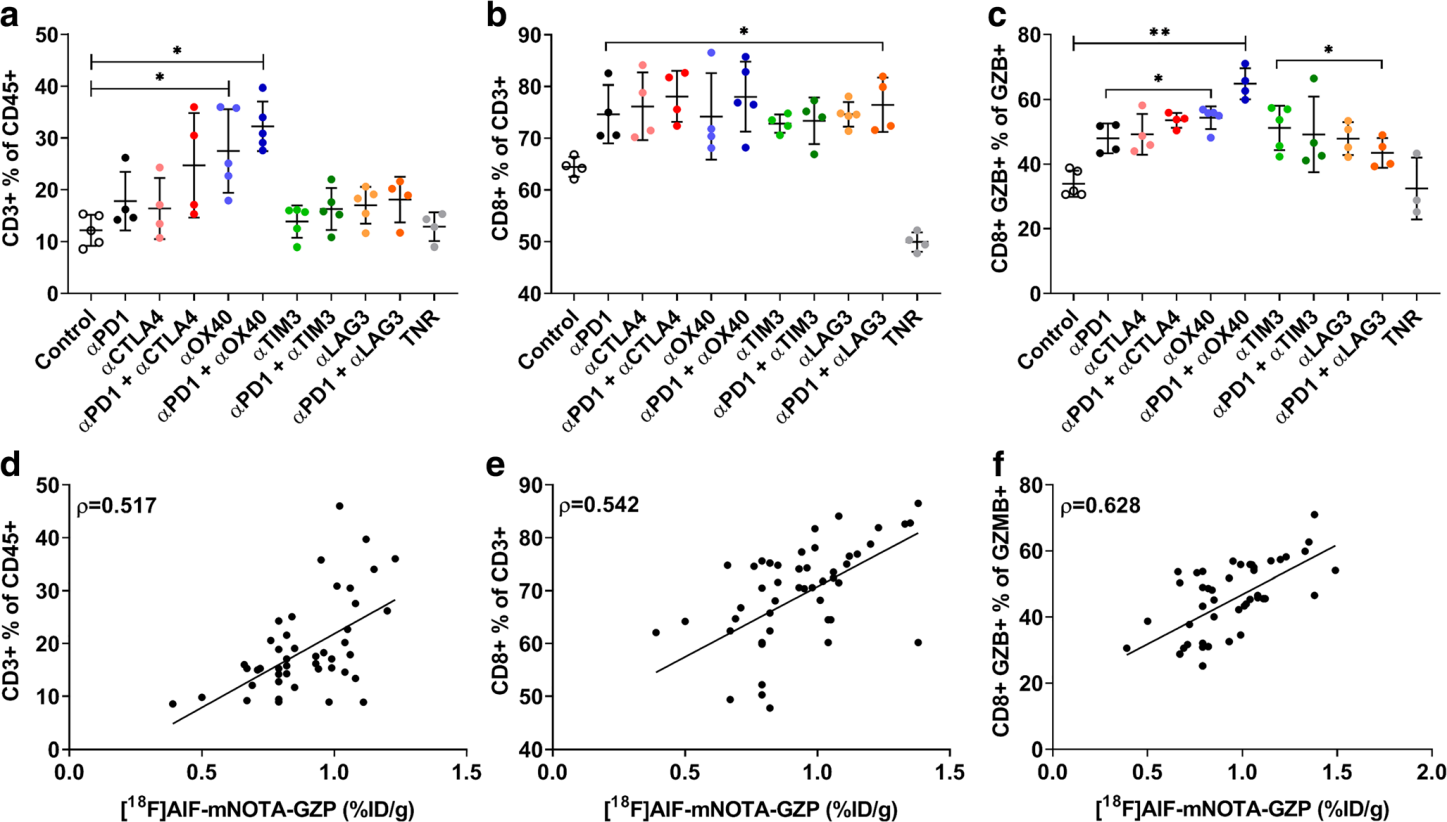
FACS analysis of tumour-infiltrating leukocyte (TIL) populations from MC38 tumour-bearing mice at day 14 post-induction of ICI monotherapy or combination therapies. Percentages of **a** CD3 T cells relative to CD45+ T cells, **b** CD8+ TILS relative to total CD3+ TILS, and **c** CD8+ GZB+ TILS relative to total GZB+ cells across all treatment arms. Data are shown as individual values with mean ± SD and are representative of *n* = 5–10 mice/group. *P < 0.05; **P < 0.01; ***P < 0.001. **d**–**f** Tumour uptake of [^18^F]AlF-mNOTA-GZP (%ID/g) plotted against paired values for relative abundance of TILs gated for **d** CD3+, **e** CD8+ and **f** CD8+ GZB cells. A linear regression model of tracer uptake *versus* individual TILs is shown, along with Spearman’s correlation (*ρ*) and corresponding *P* values. Spearman’s *ρ* show strongest correlation between [^18^F]AlF-mNOTA-GZP tumour uptake and CD8+ GZB+ cells TILs.

Changes in MC38 tumour infiltrates in response to therapy were more variable than changes in CT26 tumour infiltrates (Table 3, Fig. 5a–c). To compare, significant increases in CD3+ infiltration were observed in responders to ⍺OX40 monotherapy (29.5 ± 11.3; *P < 0.05) and ⍺PD1 + ⍺OX40 combination therapy (32.3 ± 4.8; *P < 0.05) only, compared to control treatment (12.2 ± 3.0) and TNRs (12.8 ± 2.8). No significant increases were observed after treatment with ⍺PD1 (17.8 ± 5.7), ⍺CTLA4 (16.4 ± 5.9), ⍺TIM3 (13.8 ± 3.1) or ⍺LAG3 monotherapy (17.0 ± 3.6). Likewise, no significant increases were observed after treatment with ⍺PD1 + ⍺CTLA4 (24.7 ± 10.1), ⍺PD1 + ⍺TIM3 (16.3 ± 4.1) or ⍺PD1 + ⍺LAG3 combination therapies (18.1 ± 4.4; shown as a % of CD45+ cells).

**Table 3:** FACS analysis of tumour-infiltrating leukocyte (TIL) populations from MC38 tumour-bearing mice at day 14 post-induction of ICI monotherapy or combination therapies. Percentages of T cell subpopulations across control groups, treatment responder (TR) arms, and all treatment non-responders (TNR) across all treatment arms. Data are shown as mean % of cells ± SD and are representative of *n* = 5–10 mice/ group. *P < 0.05; **P < 0.01, comparing TR to TNR

	MC38		
	CD3+ % of CD45+	CD8+ % of CD3+	CD8+ GZB+ % of GZB+
Control	12.2 ± 3.0	64.5 ± 1.9	33.9 ± 4.1
TR			
αPD1	17.8 ± 5.7	74.7 ± 5.6*	48.0 ± 4.6*
αCTLA4	16.4 ± 5.9	76.2 ± 6.5*	49.2 ± 6.3*
αPD1 + αCTLA4	24.7 ± 10.1	78.1 ± 4.9*	53.6 ± 2.3*
αOX40	29.5 ± 11.3*	74.2 ± 6.3*	54.4 ± 3.5*
αPD1 + αOX40	32.3 ± 4.8*	78.0 ± 6.7*	64.8 ± 4.7**
αTIM3	13.8 ± 3.1	72.8 ± 1.8*	51.2 ± 6.9*
αPD1 + αTIM3	16.3 ± 4.1	73.4 ± 4.5*	49.2 ± 11.7*
αLAG3	17.0 ± 3.6	74.6 ± 2.4*	47.9 ± 5.1*
αPD1 + αLAG3	18.1 ± 4.4	76.5 ± 5.2*	43.5 ± 4.6*
TNR	12.8 ± 2.8	49.9 ± 1.8	32.4 ± 9.6

CD8 infiltrates, however, were better aligned with response, showing significant increases in response to ⍺PD1 (74.7 ± 5.6; *P < 0.05), ⍺CTLA4 (76.2 ± 6.5; *P < 0.05), ⍺OX40 (74.2 ± 8.3; *P < 0.05), ⍺TIM3 (72.8 ± 1.8; *P < 0.05) and ⍺LAG3 monotherapy (74.6 ± 2.4; *P < 0.05). Significant increases were also observed after treatment with ⍺PD1 + ⍺CTLA4 (78.1 ± 4.9; *P < 0.05), ⍺PD1 + ⍺OX40 (78.0 ± 6.7; *P < 0.05), ⍺PD1 + ⍺TIM3 (73.4 ± 4.5; *P < 0.05) and ⍺PD1 + ⍺LAG3 combination therapies (76.5 ± 5.2; *P < 0.05) compared to control treatment (64.5 ± 1.9) and TNRs (49.9 ± 1.8, as a % of CD3+ cells).

CD8+ GZB+ infiltrates also showed significant increases in response to ⍺PD1 (48.0 ± 4.6; *P < 0.05), ⍺CTLA4 (49.2 ± 6.3; *P < 0.05), ⍺OX40 (54.4 ± 3.5; *P < 0.05), ⍺TIM3 (51.2 ± 6.9; *P < 0.05) and ⍺LAG3 monotherapy (47.9 ± 5.1; *P < 0.05). Significant increases were also observed after treatment with ⍺PD1 + ⍺CTLA4 (53.6 ± 2.3; *P < 0.05), ⍺PD1 + ⍺OX40 (64.8 ± 4.7; **P < 0.01), ⍺PD1 + ⍺TIM3 (49.2 ± 11.7; *P < 0.05) and ⍺PD1 + ⍺LAG3 combination therapies (43.5 ± 4.6; *P < 0.05) compared to control treatment (33.9 ± 4.1) and TNRs (32.4 ± 9.6, % of GZB+ cells).

Correlation analysis of [^18^F]AlF-mNOTA-GZP tumour uptake with TILs was performed in MC38 tumours. PET imaging showed a positive association with CD3+ T cells (Fig. 5d, *ρ* = 0.517; *P* < 0.0001), and CD8 T cells (Fig. 5e, *ρ* = 0.542; *P* < 0.0001) with the strongest correlation observed with GZB+ CD8+ T cells (Fig. 5f, *ρ* = 0.628; *P* < 0.0001).

## Discussion

Immune checkpoint inhibitor (ICI) monotherapy has been shown to trigger activation of compensatory T cell-associated checkpoints [5], such as PD-1 blockade upregulating TIM-3, an effect that could perhaps be mitigated by combined therapy. Emerging evidence supports the use of combined ICI regimens, although toxicity profiles, especially those associated with combined CTLA-4 blockade, may be of concern [3]. With the substantial number of clinical trials evaluating ICI combination therapies, the ability to rapidly and reliably assess therapy efficacy is paramount to ensure safe patient management.

Non-invasive molecular imaging of immunotherapy has predominantly focused on the expression of immunologically relevant targets within the tumour microenvironment. The majority of studies have investigated the utility of measuring PD-L1 expression to predict therapy response and have shown that while they may have excellent utility for patient selection, PD-L1 expression is dynamic and context dependent and high PD-L1 expression does not guarantee therapy response [23, 24]. Immunotherapy success is dependent on TILs and numerous biomarkers have been developed to assess changes in specific T cell subsets by the specific targeting of cell-surface CD molecule expression (CD3, CD8) [13–15]; however, high TIL expression does not guarantee therapy response either, due to tolerance and regulatory mechanisms [25, 26]. Detection of molecules that represent an effector T cell phenotype has proven to be more successful. The radiolabelled GZB targeting peptide [^68^Ga]Ga-mNOTA-GZP has been shown to be well correlated with immunotherapy efficacy [16–18]. In the current study, we have radiolabelled the GZB targeting peptide using [^18^F]AlF to improve PET imaging duration of use, sensitivity and spatial resolution [19]. The addition of [^18^F]AlF did not substantially change the enzymatic activity of the peptide (Supplemental Fig. S1), nor did it change the *in vivo* uptake and excretion kinetics compared to the ^68^Ga-labelled peptide (Supplemental Fig. S2a and b). Furthermore, when compared to our previously published data on the ^68^Ga-labelled peptide, very similar tracer uptake was observed in CT26 tumours in both ICI treatment responder and non-responder groups [18].

Interestingly, both radiopharmaceutical uptake and response to therapy was very different in the CT26 and MC38 tumours. These differences are likely due to the MSI phenotype found in MC38 tumours. MSI or deficiency of DNA mismatch repair system (MMR) occurs in approximately 15 % of colorectal cancers [20] and renders cancer cells unable to correct errors that occur at microsatellite regions during DNA replication. This leads to the accumulation of mutations which likely increase the amount and immunogenicity of neoantigens [12, 27–29]. Even with such an immunostimulatory environment, only ~ 50 % of MSI colorectal cancers respond to classical ICIs clinically while MSI negative colorectal cancers generally do not respond [20]. The effects of this immunostimulatory environment can be clearly observed in the tumour responses to ICIs and subsequent [^18^F]AlF-mNOTA-GZP uptake. Overall, the CT26 tumours showed a modest (~ 40 %) response to ⍺PD1 monotherapy and an improved (~ 70 %) response to ⍺CTLA4 or ⍺OX40 monotherapy or combined ⍺PD1 with ⍺CTLA4 or ⍺PD1 with ⍺OX40 therapy, as determined by tumour growth inhibition. The newer combination therapies ⍺TIM3 and ⍺LAG3, however, did not effectively inhibit tumour growth in the CT26 tumour bearing mice when used as monotherapies. Furthermore, neither ⍺TIM3 or ⍺LAG3, effectively inhibited tumour growth when used in combination with ⍺PD1, despite ⍺PD1 monotherapy proving efficacious in 40 % of tumours when used as a monotherapy. This lack of response may be due to the relatively low total number of CT26 resident cytotoxic T cells, despite tumour-infiltrating CD8+ cells having been shown to express TIM-3 and LAG-3 [19, 30]. The low number of T cells associated with the CT26 tumours leads to a relatively low tumour uptake of [^18^F]AlF-mNOTA-GZP at ~ 0.2%ID/g in non-responsive tumours, peaking at ~ 0.6%ID/g in treatment responsive tumours. The MC38 tumours, with a much greater number of cytotoxic T cells, displayed a higher response rate to all therapies (~ 70–90 % response to ⍺PD1, ⍺CTLA4, ⍺OX40 or combinations and successfully responded to ⍺TIM3, ⍺LAG3 monotherapies or combinations (~ 40–60 %). [^18^F]AlF-mNOTA-GZP uptake was similarly affected by the immunostimulatory environment, with significantly higher uptake in all the MC38 tumours (in controls, TRs and TNRs alike) ranging from ~ 0.6%ID/g in non-responsive tumours (levels comparable to peak responders in the CT26 tumour type) and up to ~ 1.1%ID/g in treatment-responsive tumours. In both tumour types, uptake of [^18^F]AlF-mNOTA-GZP in TRs were positively correlated with CD3+, CD8+ and CD8+GZB+ TILs (Figs. 4 and 5), however, the variable levels of immune cells prevalent in the MSI high MC38 tumours, obscured the changes in T cell populations diminishing the correlation between TILs and [^18^F]AlF-mNOTA-GZP uptake when compared to the MSI-low CT26 tumours. The difference in tumour immunostimulatory environments may be a limiting factor affecting the utility of [^18^F]AlF-mNOTA-GZP, potentially obfuscating response stratification in immune-sensitive tumours.

Furthermore, while the number of non-responsive MC38 tumours was relatively low, those that did not respond displayed an intriguing reduction in CD8+ TILs compared with the control treated group, potentially linked with the regulatory suppression mechanisms that caused these tumours to be non-responsive. While a small increase in CD4+ FOXP3+ CD25+ Tregs was observed in the MC38 TNRs when compared to the control treated tumours this was not significant. Certainly, the presence of mutations can influence the function of several cellular processes and these may include those that are designed to nullify the host response.

## Conclusions

In summary, the current study shows that *in vivo* molecular imaging of intra-tumoural granzyme B using [^18^F]AlF-mNOTA-GZP is able to stratify tumour response to a range of ICIs administered to preclinical colon cancer models given as monotherapies or in combination. However, care should be taken in interpreting the images, as tracer uptake can be significantly affected by pre-existing phenotypic abnormalities. Further studies will be needed to elucidate other factors affecting the interpretation of granzyme B targeting tracers before they could be used for stratification of patient response across multiple tumour phenotypes.

## Electronic Supplementary Material

ESM 1 (PNG 71 kb)

High Resolution Image (TIF 163 kb)

ESM 2 (PNG 379 kb)

High Resolution Image (TIF 731 kb)

ESM 3 (PNG 49 kb)

High Resolution Image (TIF 155 kb)

ESM 4 (PNG 1306 kb)

High Resolution Image (TIF 5663 kb)

ESM 5 ( 31 kb)
