# Supplementary figures and images for: Granzyme B PET Imaging of Immune Checkpoint Inhibitor Combinations in Colon Cancer Phenotypes
Source: Mol Imaging Biol. 2020 Jul 23;22(5):1392–402. doi: 10.1007/s11307-020-01519-3 (PMC7497445; doi:10.1007/s11307-020-01519-3)

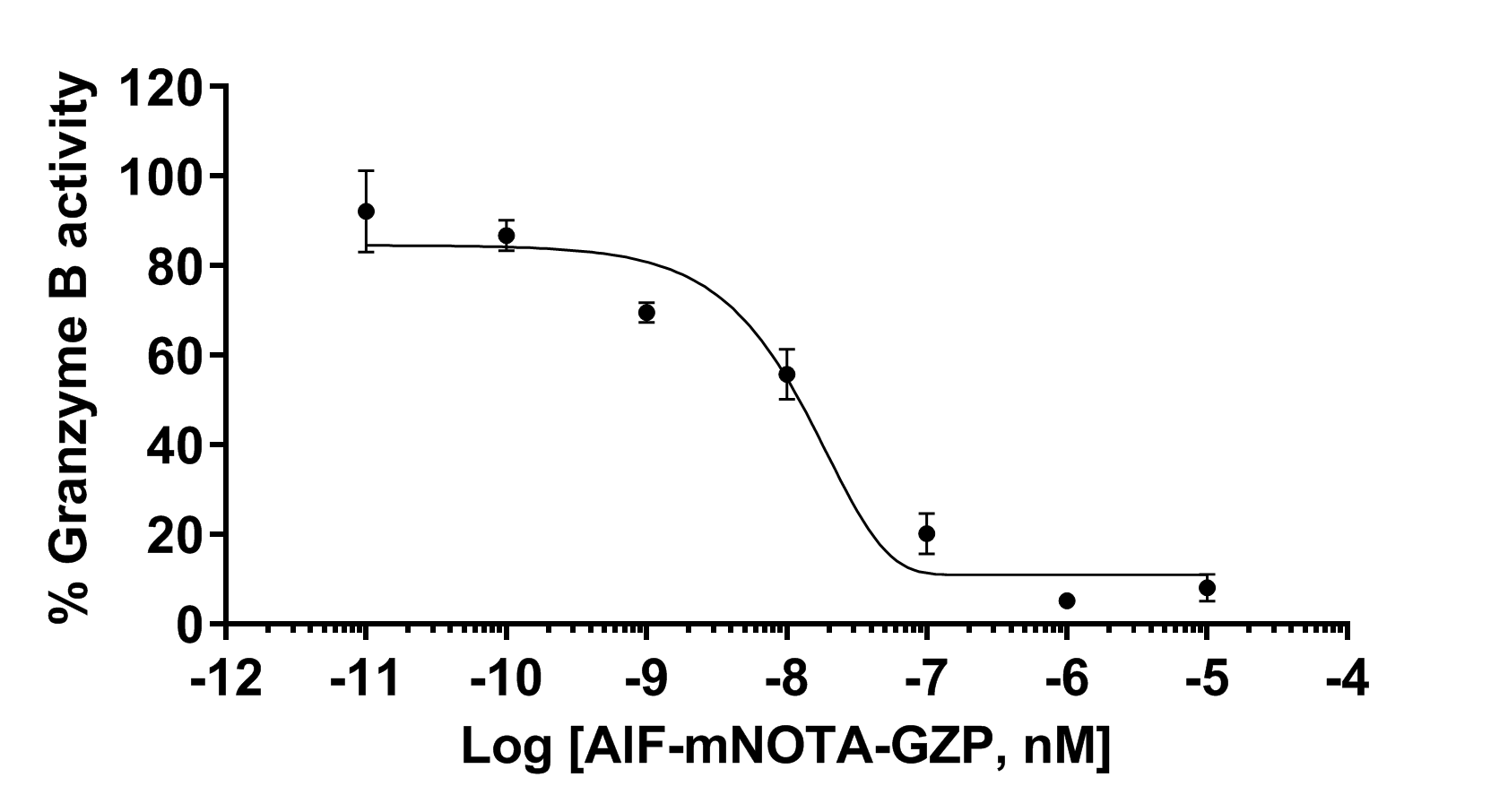

Supplement: Supplementary file 1 — (PNG 71 kb) [file 11307_2020_1519_Fig6_ESM.png]

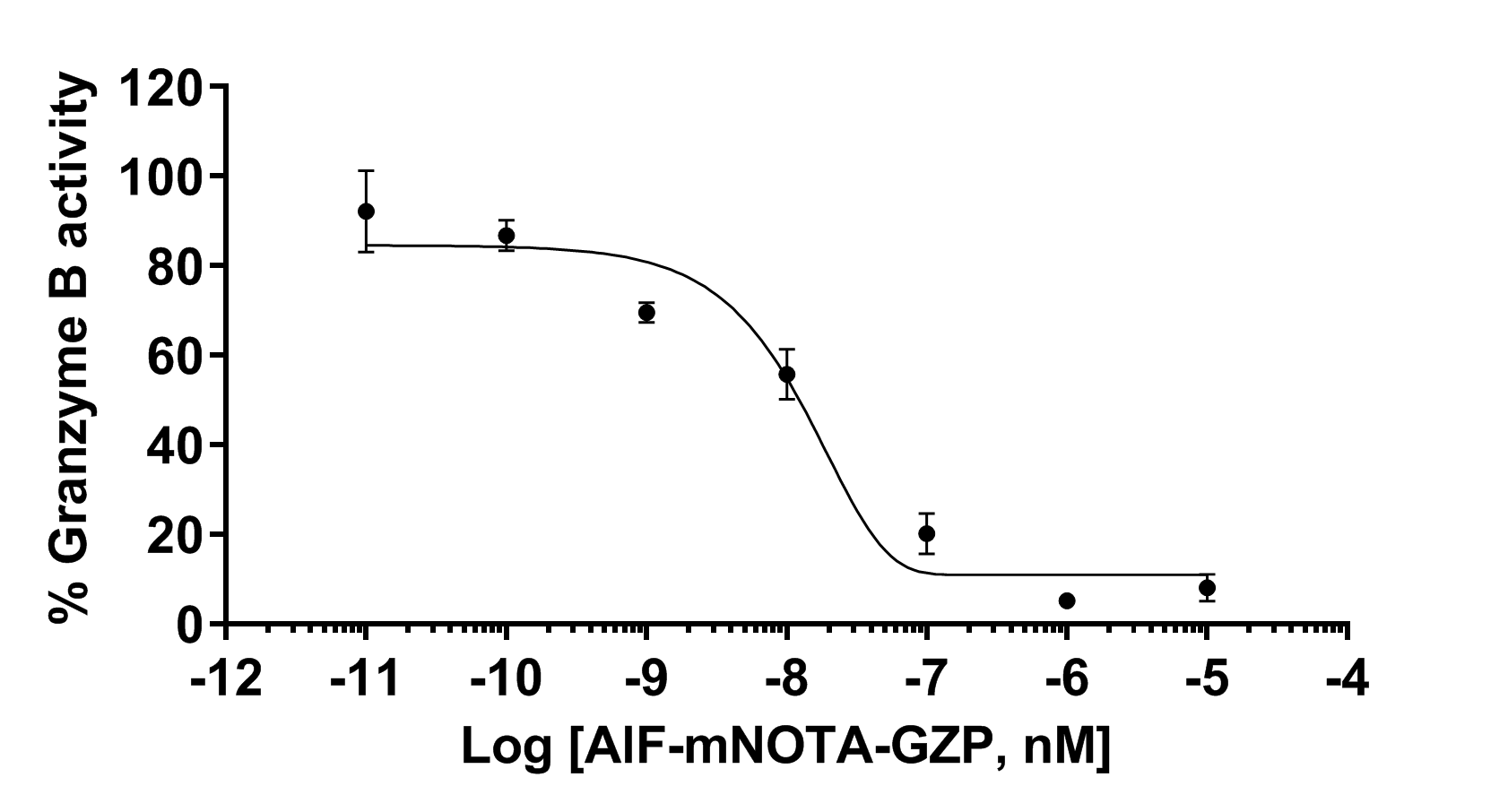

Supplement: Supplementary file 2 — High Resolution Image (TIF 163 kb) [file 11307_2020_1519_MOESM1_ESM.tif]

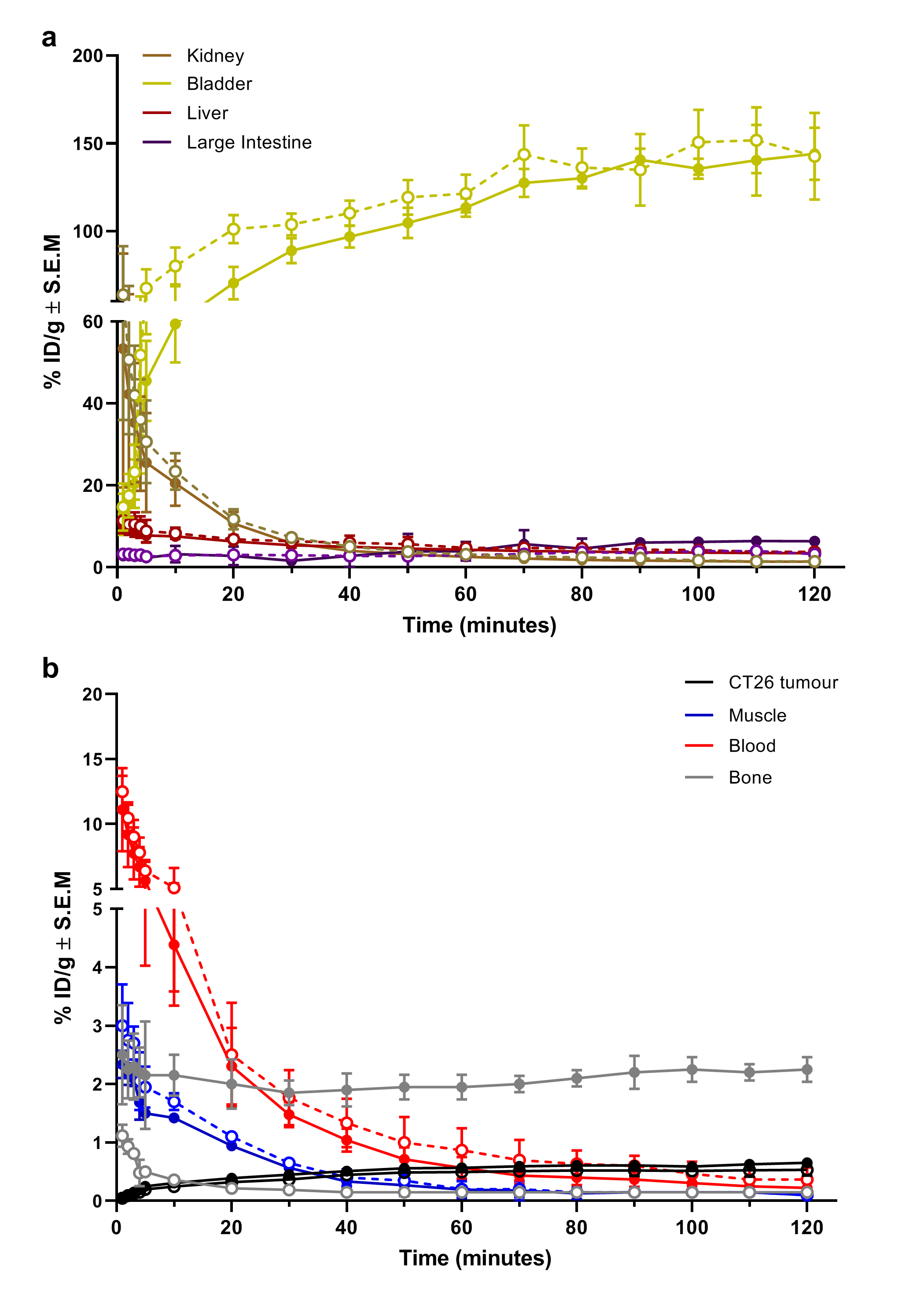

Supplement: Supplementary file 3 — (PNG 379 kb) [file 11307_2020_1519_Fig7_ESM.png]

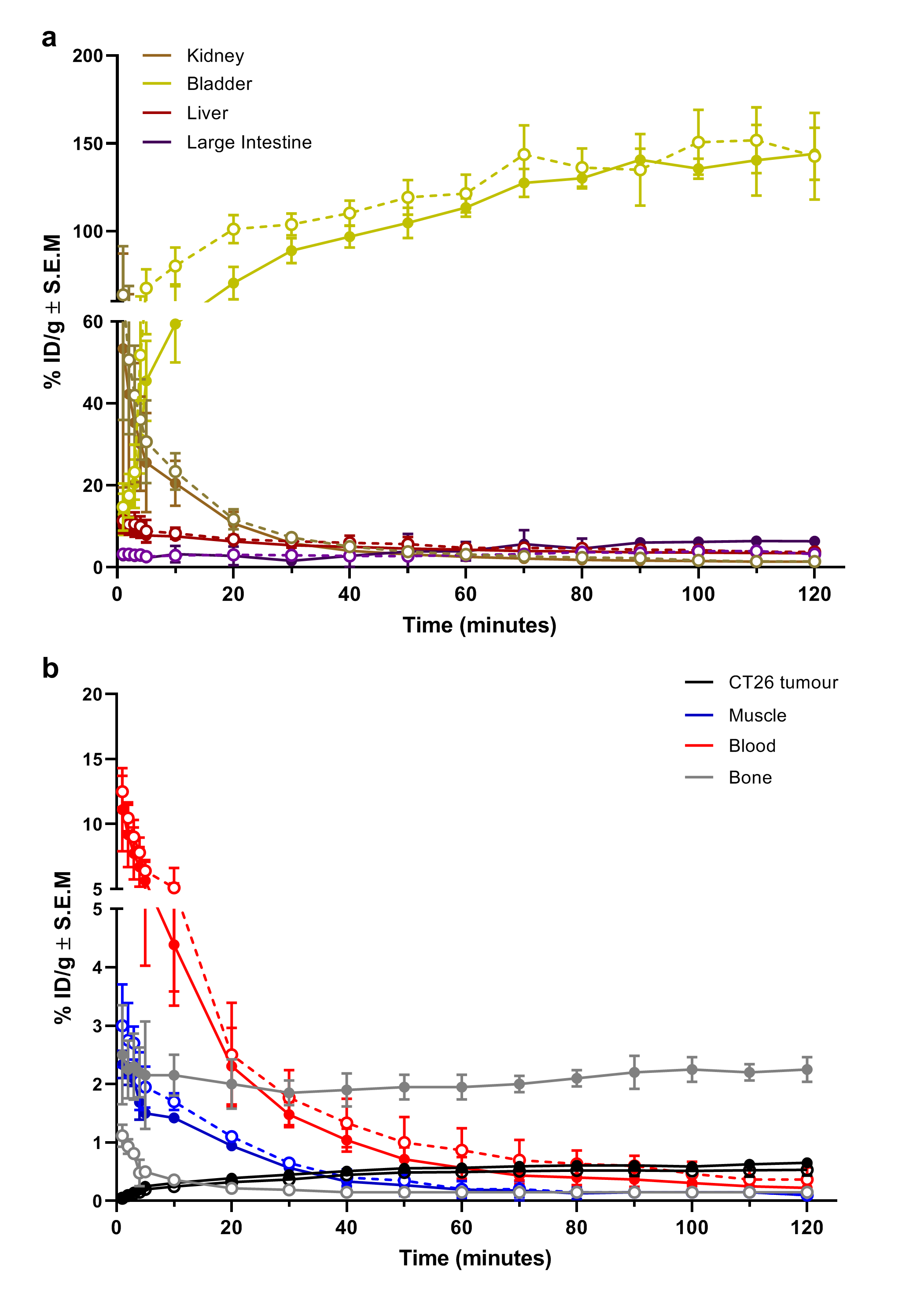

Supplement: Supplementary file 4 — High Resolution Image (TIF 731 kb) [file 11307_2020_1519_MOESM2_ESM.tif]

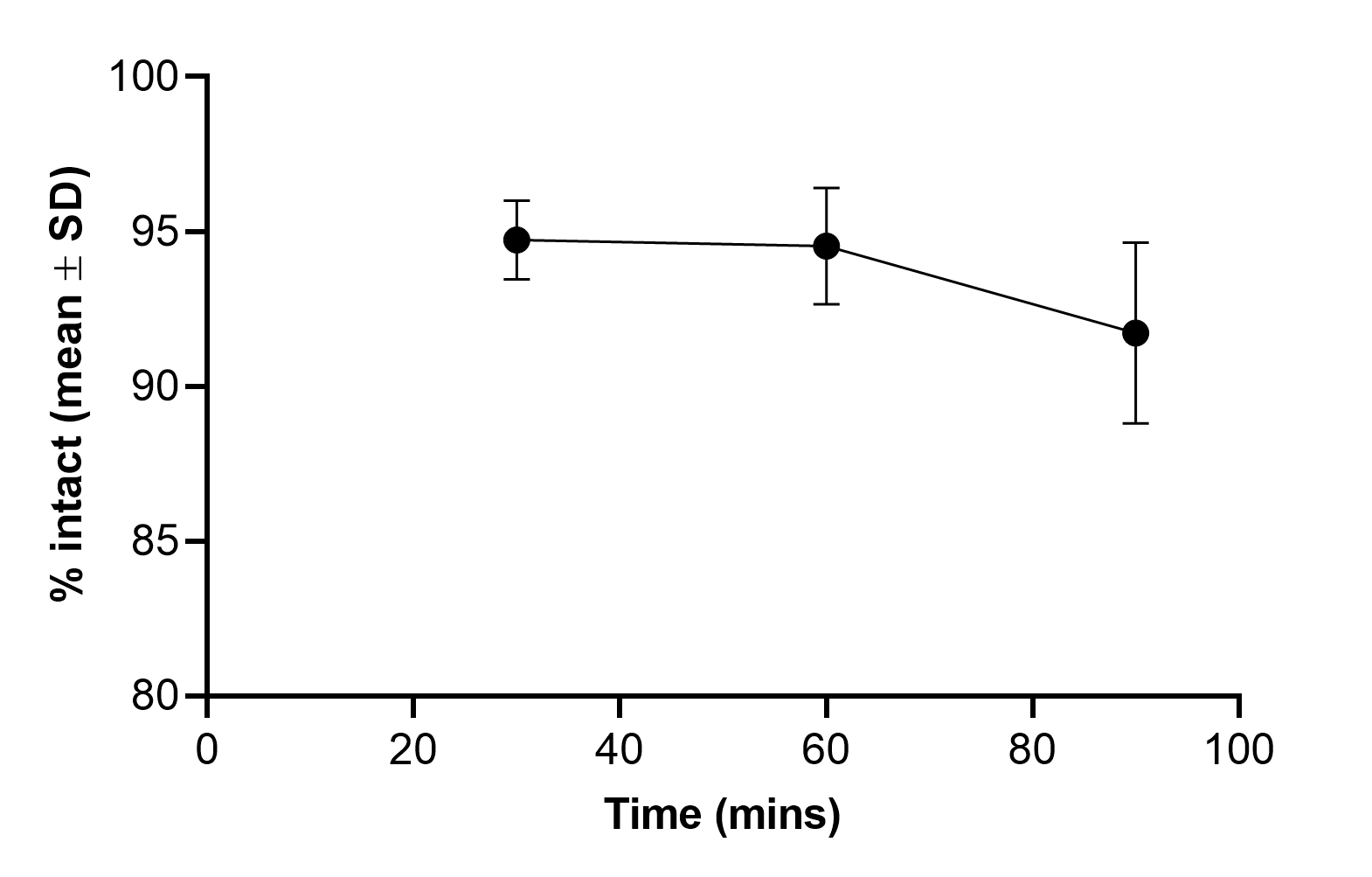

Supplement: Supplementary file 5 — (PNG 49 kb) [file 11307_2020_1519_Fig8_ESM.png]

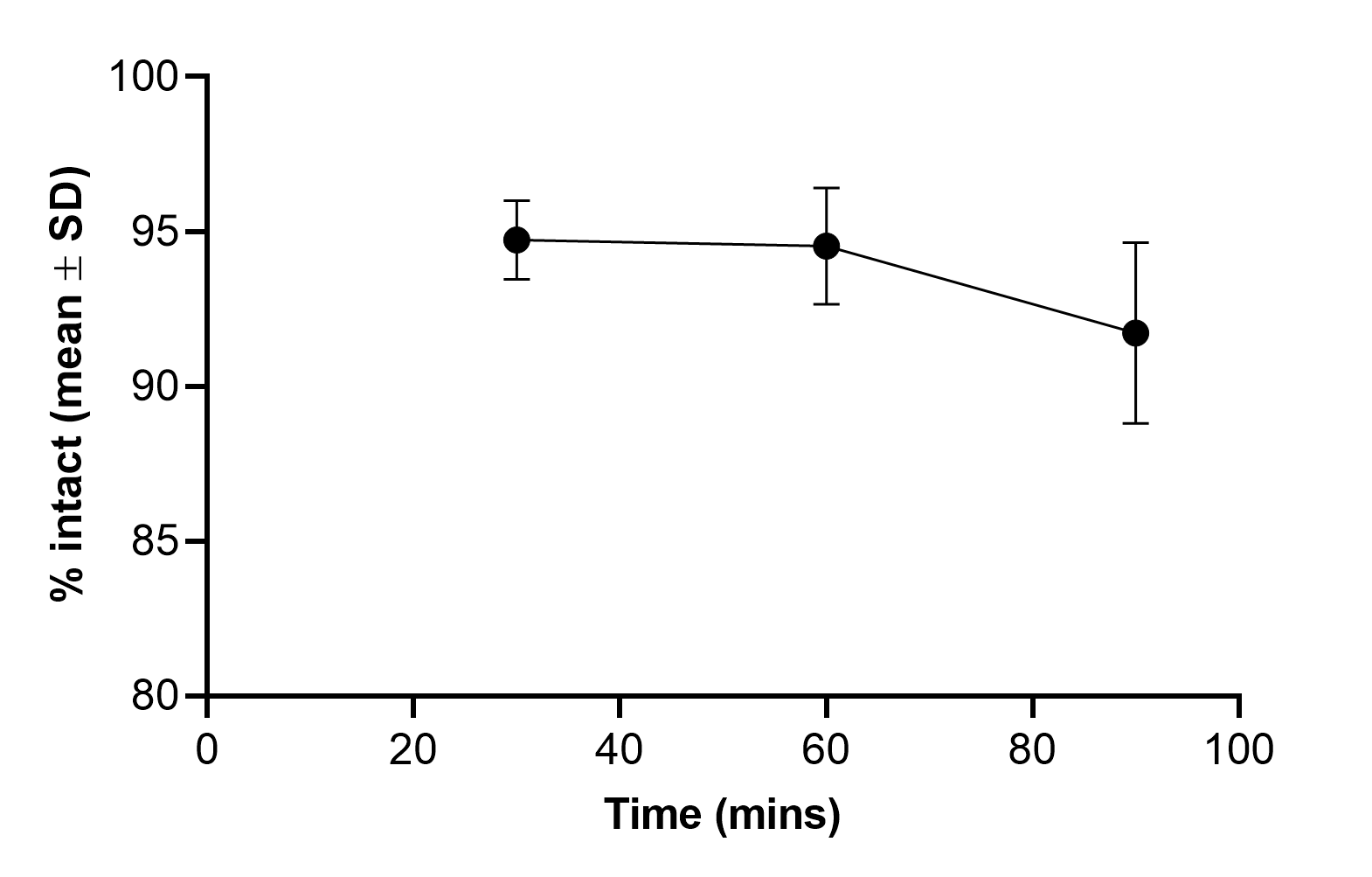

Supplement: Supplementary file 6 — High Resolution Image (TIF 155 kb) [file 11307_2020_1519_MOESM3_ESM.tif]

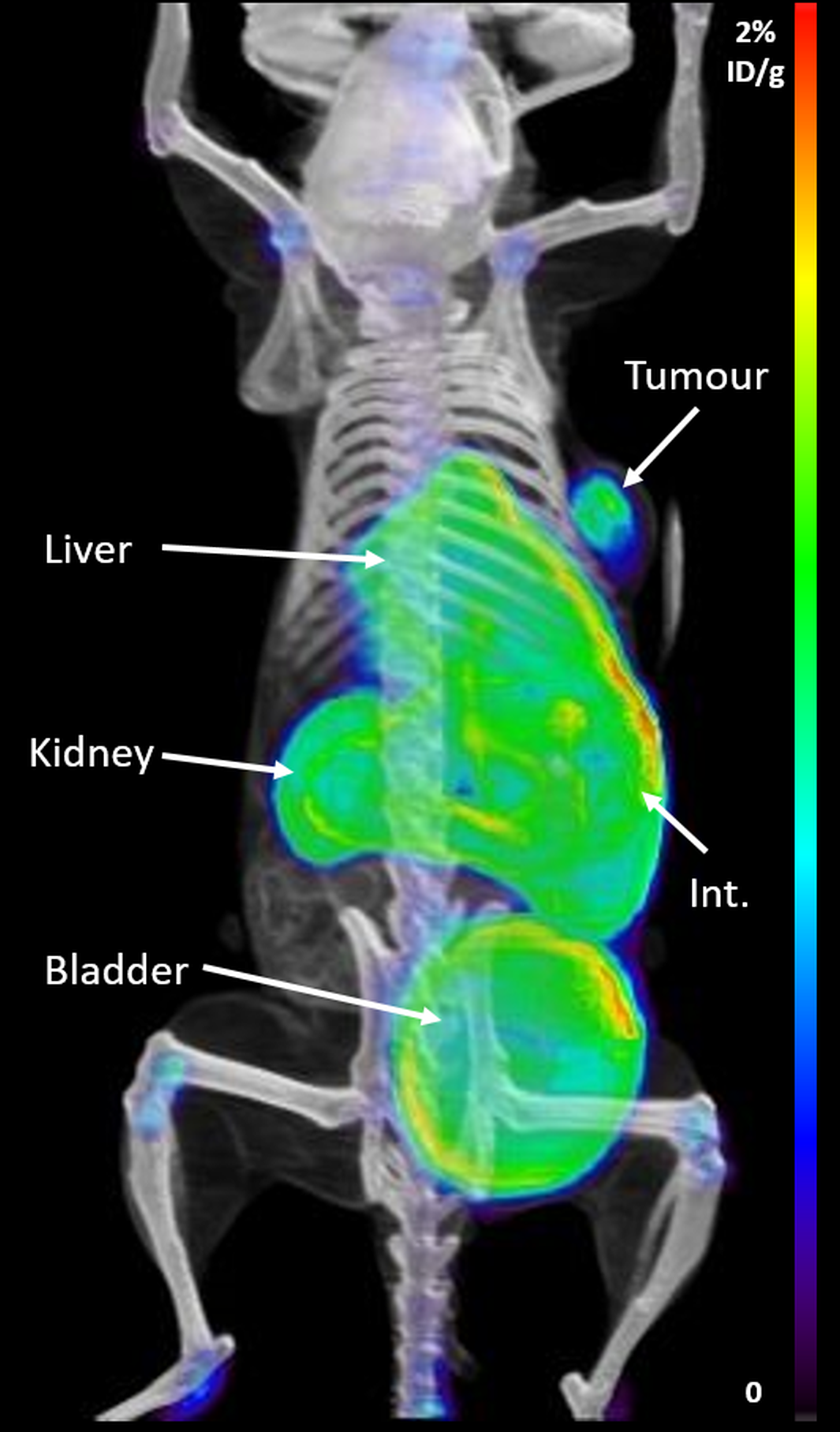

Supplement: Supplementary file 7 — (PNG 1306 kb) [file 11307_2020_1519_Fig9_ESM.png]

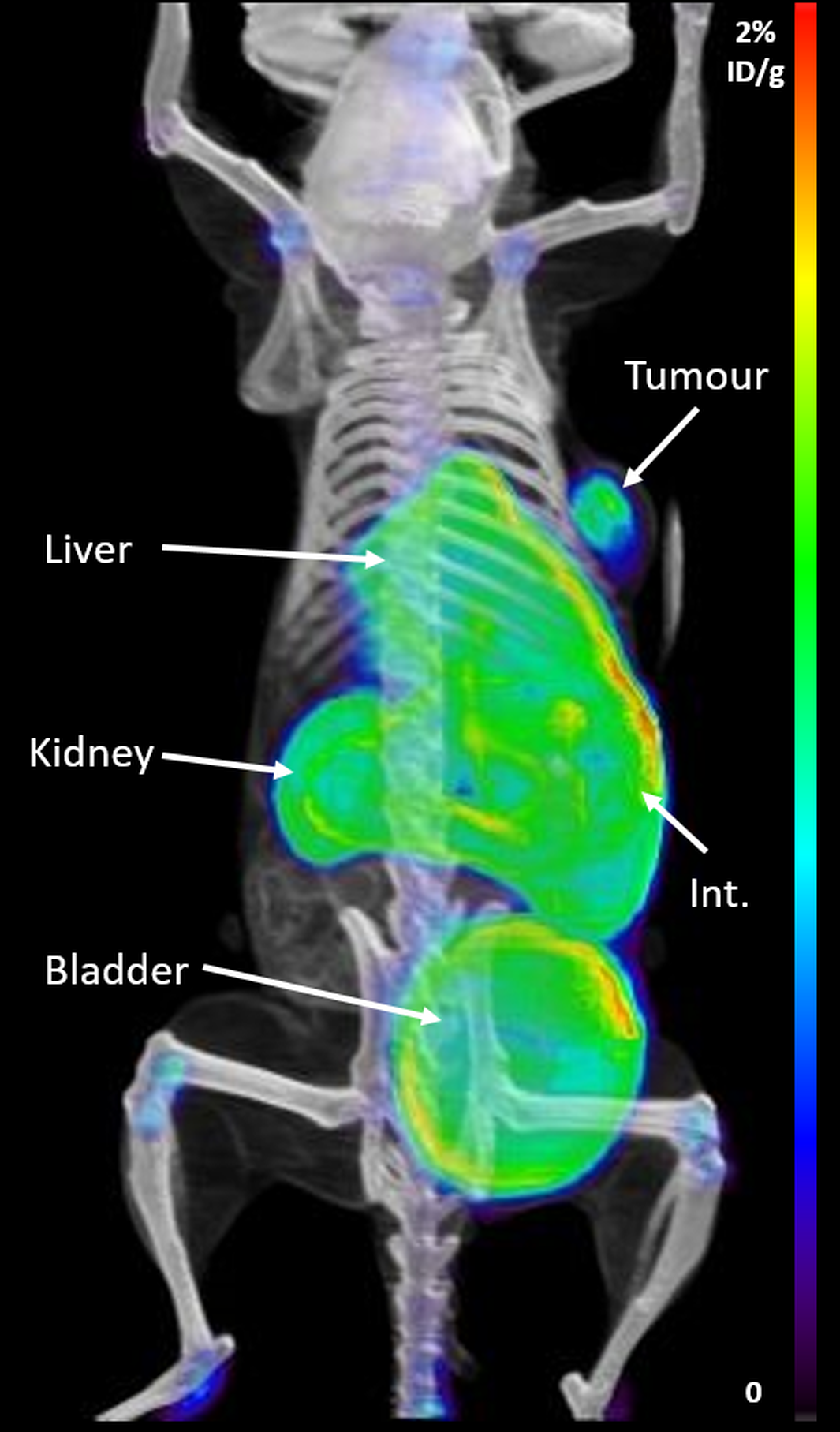

Supplement: Supplementary file 8 — High Resolution Image (TIF 5663 kb) [file 11307_2020_1519_MOESM4_ESM.tif]
