# Supplementary material for: Granzyme B PET Imaging of Immune Checkpoint Inhibitor Combinations in Colon Cancer Phenotypes
Source: Mol Imaging Biol. 2020 Jul 23;22(5):1392–402. doi: 10.1007/s11307-020-01519-3 (PMC7497445; doi:10.1007/s11307-020-01519-3)
Supplement: Supplementary file 9 — ( 31 kb) [file 11307_2020_1519_MOESM5_ESM.docx]

# SUPPLEMENTAL

# Granzyme B PET imaging of immune checkpoint inhibitor combinations in colon cancer phenotypes.

Goggi JL^a^; Tan YX^a^; Hartimath SV^a^; Jieu B^d^; Hwang YY^b^; Jiang L^a^; Boominathan R^a^; Cheng P^a^; Yuen TY^d^; Chin HX^b^; Tang JR^a^; Larbi A^b^; Chacko AM^e^; Renia L^b^; Johannes C^c^, Robins EG^a,f*^

^a^ Singapore Bioimaging Consortium, Agency for Science, Technology and Research (A* STAR), 11 Biopolis Way, #01-02 Helios, Singapore, 138667

^b^ Singapore Immunology Network, A*STAR, 8A Biomedical Grove, Immunos, Singapore, 138648

^c^ p53 Laboratory, A*STAR, 8A Biomedical Grove, #06-04/05, Neuros/Immunos, Singapore 138665

^d^ Institute of Chemical and Engineering Sciences (ICES), A*STAR, 8 Biomedical Grove, #07, Neuros, Singapore 138665

^e^ Laboratory for Translational and Molecular Imaging (LTMI), Cancer and Stem Cell Biology Programme, Duke-NUS Medical School, 8 College Road, Singapore 169857

^f^ Clinical Imaging Research Centre (CIRC), Yong Loo Lin School of Medicine, National University of Singapore, Singapore, 117599

*Corresponding author:

Dr Edward G Robins

[edward_robins@sbic.a-star.edu.sg](mailto:edward_robins@sbic.a-star.edu.sg)

Tel: +65 6478 7001

Fax: +65 6478 8908

Abbreviated title: Imaging immunotherapy combinations.

**Supplemental Methods**

**1.1 General information**

H-Asp(OtBu)-H NovaSyn TG resin (0.21 mmol g^-1^) was obtained from Merck (Singapore). Fmoc-amino acids, Hexafluorophosphate Azabenzotriazole Tetramethyl Uronium (HATU) and 1-Hydroxy-7-azabenzotriazole (HOAt) were obtained from Advanced Chemtech (Louisville, KY). Fmoc-glutamic acid was t-butyl protected. (p-SCN-Bn)-NOTA was purchased from Boc Sciences (New York, United States) and Macrocyclics (New York, United States). Trifluoroacetic acid (TFA) and N, N-Diisopropylethylamine (DIPEA) was purchased from Tokyo Chemical Industry (Tokyo, Japan) while all other solvents and reagents were obtained from Fisher Scientific (Loughborough, United Kingdom). All reagents were used as received.

**1.2 Peptide Synthesis**

*Solid-phase peptide synthesis.* The murine Granzyme B (mGZP) peptide (βA)GGIEFD-CHO was synthesised manually using Fmoc chemistry on H-Asp(OtBu)-H NovaSyn TG resin (0.21 mmol g^-1^). The dry resin was weighed out into a fritted reaction vessel and swelled with DMF for 60 min before use. With the exception of aspartic acid, which was pre-loaded and unprotected at the N-terminus, the Fmoc protecting group on amino acids were removed by treatment with 20% piperidine in DMF and argon gas bubbling (30 min). Fmoc-protected amino acids (4 equiv.) were coupled using pre-activated (7 min) solutions of HATU (4 equiv.), HOAt (4 equiv.) and DIPEA (8 equiv.) in NMP (0.2 M) for 60 min. A Kaiser test was done to determine coupling completion and amino acids were double coupled when necessary. The resin was thoroughly washed with DMF after each coupling and deprotection reaction.

*(p-SCN-Bn)-NOTA conjugation and cleavage.* Following assembly of the peptide, the final Fmoc group was deprotected with 20% piperidine in water for 30 min. (p-SCN-Bn)-NOTA (1.5 equiv.) was pre-activated (7 min) with DIPEA (4 equiv.) in NMP (0.1 M) and coupled onto the N-terminal residue for two hours. The *t*-butyl protecting group was cleaved from glutamic acid with two rounds of treatment with TFA for ten minutes. Cleavage of the peptide from the resin was achieved using a cocktail of acetic acid:water:DCM:DMF (10:5:64:21) (3 x 30 min). The peptide solution was collected by filtration, diluted in a mixture of acetonitrile and water (1:3) and lyophilised.

*Peptide purification and analysis.* The dried peptide was re-dissolved in acetonitrile and water (1:3) and purified *via* reverse-phase HPLC using an Agilent 1260 Infinity system fitted with a Phenomenex^®^ preparative column (Jupiter C12, 4 µm, Proteo 90 Å, 250 x 10 mm). Eluents used were 0.1% aqueous TFA and 0.1% TFA in acetonitrile and water (9:1). Peptide purity and molecular weight (Table S1) were confirmed *via* UPLC-MS using an Agilent 1260 Infinity II system fitted with a Phenomenex® analytical column (Aeris 1.7 µm, Peptide XB-C18 100 LC Column, 150 x 2.1 mm). Eluents used were 0.1% aqueous formic acid and 0.1% formic acid in acetonitrile and water (9:1).

Table S1. Mass spectrometry data of NOTA-mGZP

| **Peptide** | **Sequence** | **m/z (calculated)** | **m/z (observed)** |
| --- | --- | --- | --- |
| NOTA-mGZP | NOTA-(βA)GGIEFD-CHO | 1141 | 1142 [M+H], 571 [M/2+H] |

**1.3 Synthesis of [^19^F]AlF-mNOTA-GZP**

To a solution of mNOTA-GZP (0.5 mg, 0.44 µmol) in ethanol (1.65 mL) was added 2 mM AlCl_3_ in 0.1 M pH 4 NaOAc buffer (0.55 mL, 1.1 µmol, 2.5 eq.) and 2 mM NaF in 0.1 M pH 4 NaOAc buffer (1.1 mL, 2.2 µmol, 5.0 eq.). The reaction vial was sealed and heated at 100 °C for 15 min without stirring. After cooling to room temperature, the crude reaction mixture was diluted with water (15 mL) and loaded on a pre-conditioned Sep-Pak^®^ C18 light cartridge. The cartridge was washed with a further 5 mL of water, followed by elution in fractions of 70% ethanol in water (4 x 0.1 mL). The most concentrated fraction (vial 3) was diluted with a further 0.6 mL of 0.9% w/v saline to give a solution of [^19^F]AlF-mNOTA-GZP (0.25 mg/mL, calculated based on a calibration curve) in 10% ethanol in saline. The final product was characterised by analytical HPLC (Luna Phenomenex C18(2), 5µm, 100 Å, 250 mm x 4.6, 0.05M NH_4_OAc pH 4.5 (solvent A) and acetonitrile (solvent B), gradient elution 0–6 minutes 10% to 95% B then 6–11 minutes 95% B, flow rate 1 mL/min, column temperature 30°C, λ = 254 nm). The retention time of [^19^F]AlF-mNOTA-GZP was between 7.1 to 7.2 minutes. Aluminium fluoride chelation was confirmed by LCMS, calculated [M-H]- 1184.43, found 1184.35.

**1.4 *In vitro* assessment of mNOTA-GZP**

As Granzyme B (GZB) is a serine protease stored in the granules of activated cytotoxic CD8+ T cells, we confirmed the ability of AlF-mNOTA-GZP to inhibit the enzymatic cleavage of GZB synthetic substrate BOC–Ala–Ala–Asp–SBZL, as previously described [[1](#_ENREF_1)]. AlF-mNOTA-GZP ligand maintained high inhibitory potency of K_i_ ~78 ± 36 nM (Figure S1).

**1.5 *In vivo* biodistribution of [^18^F]AlF-mNOTA-GZP compared to [^68^Ga]Ga-mNOTA-GZP**

The biodistribution of [^18^F]AlF-mNOTA-GZP was compared to [^68^Ga]Ga-mNOTA-GZP in BalbC mice bearing CT26 tumours treated with combined PD-1 and CTLA4 therapy. The animals were imaged dynamically for 120minutes and the distribution and excretion characteristics of the two radiopharmaceuticals compared. Overall the excretion profiles for the two radiopharmaceuticals are very similar showing rapid excretion via the kidneys and little hepatobiliary clearance (Figure S2A). Blood clearance, muscle uptake and tumour uptake are likewise similar between the radiopharmaceuticals with bone uptake the only significantly different parameter due to the propensity of free fluoride to enter bone.

**1.6 Ex *vivo* metabolite analysis of [^18^F]AlF-mNOTA-GZP**

After intravenous injection of ~20MBq [^18^F]AlF-mNOTA-GZP, blood samples (~300 µL) were collected via the orbital plexus into heparinized polypropylene centrifuge tubes, at 10, 30, 60 and 90 min p.i. Blood samples were centrifuged at 2,500 x g for 10 min. An equal amount of acetonitrile was added to the supernatant plasma samples and vortexed to precipitate out remaining proteins. The samples were centrifuged at 2,500 x g for 5 min at 4 °C, and the supernatant analyzed via radioHPLC. [^18^F]AlF-mNOTA-GZP was shown to be stable in vivo, displaying less than 10% metabolism over 90 minutes as shown in Figure S3.

Table S2. Summary of ICI treatment responders (TR) and treatment non-responders (TNR) across all therapy arms in syngeneic CT26 and MC38 colon cancer models

|  | **Treatment Responders (TR)/**  **Treatment Non-Responders (TNR)**  **(No. mice)** | |
| --- | --- | --- |
| **ICI Treatment** | **CT26** | **MC38** |
| Control | 0/10 | 0/10 |
| αPD1 | 6/15 | 7/10 |
| αCTLA4 | 6/10 | 8/10 |
| αPD1 + αCTLA4 | 8/10 | 9/10 |
| αOX40 | 7/10 | 5/6 |
| αPD1 + αOX40 | 7/10 | 5/6 |
| αTIM3 | 0/6 | 3/5 |
| αPD1 + αTIM3 | 0/5 | 3/5 |
| αLAG3 | 0/6 | 3/6 |
| αPD1 + αLAG3 | 0/6 | 2/5 |
| % Monotherapy Response | 40.4 | 70.3 |
| % Combined Therapy Response | 48.4 | 73.1 |
| % Overall Therapy Response | 43.5 | 71.4 |

**Table S3.** Summary of tumour volumes in controls, ICI treatment responders (TR) and treatment non-responders (TNR) across all therapy arms in syngeneic CT26 and MC38 colon cancer models

| **Treatment arm** | **Days post inoculation** | **CT26 tumour volume**  **(mm^3^ ± SD)** | **MC38 tumour volume**  **(mm^3^ ± SD)** |
| --- | --- | --- | --- |
| **Control** | 5  8  12  14 | 95.2 ± 6.6  191.6 ± 40.3  347.1 ± 22.3  516.9 ± 69.7 | 88.1 ± 12.9  130.1 ± 8.5  231.3 ± 22.7  488.3 ± 97.2 |
| **Treatment Responders (TR)**  **αPD1** | 5  8  12  14 | 97.0 ± 4.4  118.0 ± 16.6  202.7 ± 54.1  242.6 ± 18.0 | 93.9 ± 3.6  150.4 ± 4.4  156.4 ± 32.8  175.3 ± 33.8 |
| **αCTLA4** | 5  8  12  14 | 108.6 ± 4.6  158.5 ± 37.3  126.8 ± 16.1  96.2 ± 6.5 | 92.3 ± 7.8  117.2 ± 8.9  104.4 ± 24.9  137.8 ± 78.0 |
| **αPD1 + αCTLA4** | 5  8  12  14 | 81.7 ± 3.7  123.4 ± 12.9  154.2 ± 28.6  134.5 ± 36.2 | 91.3 ± 4.5  87.3 ± 28.5  110.4 ± 17.9  97.3 ± 33.7 |
| **αOX40** | 5  8  12  14 | 92.4 ± 10.4  148.8 ± 52.0  154.7 ± 77.7  126.5 ± 73.9 | 85.8 ± 11.4  86.0 ± 4.9  108.1 ± 14.2  105.0 ± 38.3 |
| **αPD1 + αOX40** | 5  8  12  14 | 91.5 ± 11.4  192.4 ± 30.3  202.4 ± 52.4  195.8 ± 97.4 | 87.2 ± 11.3  91.2 ± 11.6  94.9 ± 25.2  121.2 ± 32.6 |
| **αTIM3** | 5  8  12  14 | 91.3 ± 9.0  206.0 ± 19.7  346.6 ± 48.3  514.2 ± 74.1 | 100.0 ± 5.0  144.5 ± 29.6  181.0 ± 38.5  206.9 ± 85.2 |
| **αPD1 + αTIM3** | 5  8  12  14 | 91.8 ± 9.8  193.1 ± 26.1  346.5 ± 57.0  494.7 ± 119.2 | 99.2 ± 8.5  122.1 ± 26.8  164.2 ± 28.9  189.1 ± 63.7 |
| **αLAG3** | 5  8  12  14 | 91.5 ± 9.4  193.7 ± 13.8  307.1 ± 64.4  464.1 ± 184.8 | 94.7 ± 20.2  179.7 ± 41.9  200.8 ± 61.9  242.5 ± 67.1 |
| **αPD1 + αLAG3** | 5  8  12  14 | 91.6 ± 10.5  192.1 ± 42.8  341.6 ± 77.5  469.6 ± 132.4 | 89.6 ± 6.5  126.7 ± 18.2  167.1 ± 26.5  245.3 ± 95.2 |
| **Treatment Non-Responders (TNR)** | 5  8  12  14 | 97.2 ± 20.4  193.4 ± 21.0  402.5 ± 96.0  538.2 ± 194.1 | 91.1 ± 7.2  150.3 ± 22.8  186.4 ± 44.3  384.9 ± 28.7 |

**Figure Legends**

**Figure S1.** Representative *in vitro* Granzyme B enzymatic activity curve with increasing concentrations of AlF-mNOTA-GZP. Each point is the mean of 6 replicates ± S.D. Nonlinear regression curve was fitted to each independent assay (n=3), with calculated K_i_ of 74 ± 36 nM for AlF-mNOTA-GZP relative to synthetic substrate analogue.

**Figure S2.** Representative time activity curves (TACs) showing the biodistribution of [^18^F]AlF-mNOTA-GZP (full circles, solid lines) and [^68^Ga]Ga-mNOTA-GZP (open circles, dashed lines) in CT26 tumour bearing animals treated with αPD1+CTLA4 (n=4). **A.** Shows TACs highlighting the excretion of the radiolabelled peptides. **B.** Shows TACs highlighting tumour, blood, muscle and bone uptake.

**Figure S3.**

Graph showing % intact parent [^18^F]AlF-mNOTA-GZP in plasma (data shown as % intact parent ± SD).

**Figure S4.**

Representative maximum intensity projection PET/CT images of [^18^F]AlF-mNOTA-GZP tumour uptake in an MC38 tumour bearing animal showing whole body distribution for reference (white arrows show tumour, liver, kidney, bladder and intestinal uptake). Mice administered ~10 MBq [^18^F]AlF-mNOTA-GZP, and images acquired from 60-80 mins post tracer injection.

**REFERENCES**

1. Larimer BM, Wehrenberg-Klee E, Dubois F, et al. (2017) Granzyme B PET Imaging as a Predictive Biomarker of Immunotherapy Response. Cancer research 77:2318-2327.
